# Supplementary material for: Estimating excess bound water content due to serpentinisation in mature slow-spreading oceanic crust using Vp/Vs
Source: Nat Commun. 2025 Jul 23;16:6772. doi: 10.1038/s41467-025-62052-x (PMC12287309; doi:10.1038/s41467-025-62052-x)
Supplement: Supplementary file 2 — Description of Additional Supplementary Information [file 41467_2025_62052_MOESM2_ESM.pdf]

Description of Additional Supplementary Information

Supplementary Data 1. **Final 2D P-wave velocity (Vp) grid**. The grid file is netCDF format (32-bit float), which can be processed using tools such as Generic Mapping Tool (GMT) [1]. The grid spans 384 km horizontally (0-384 km, 5121 nodes with a spacing of 0.075 km) and 25 km vertically (0-25 km, 2501 nodes with a spacing of 0.01 km). Note that the Vp grid is masked by S-wave ray coverage as shown in Fig. 2a.

Supplementary Data 2. **Final 2D S-wave velocity (Vs) grid**. The grid file is in netCDF format (32-bit float), which can be processed using tools such as GMT [1]. The grid spans 384 km horizontally (0-384 km, 5121 nodes with a spacing of 0.075 km) and 25 km vertically (0-25 km, 2501 nodes with a spacing of 0.01 km). Note that the Vs grid is masked by S-wave ray coverage as shown in Fig. 2b.

Supplementary Data 3. **Final Vp/Vs ratio grid**. The grid file is in netCDF format (32-bit float), which can be processed using tools such as GMT [1]. The grid spans 384 km horizontally (0-384 km, 5121 nodes with a spacing of 0.075 km) and 25 km vertically (0-25 km, 2501 nodes with a spacing of 0.01 km). Note that the Vp/Vs ratio grid is masked by S-wave ray coverage as shown in Fig. 2c.

References

1 Wessel, P. *et al.* The Generic Mapping Tools Version 6. *Geochemistry, Geophysics, Geosystems* **20**, 5556-5564 (2019).
