# Supplementary material for: Estimating excess bound water content due to serpentinisation in mature slow-spreading oceanic crust using Vp/Vs
Source: Nat Commun. 2025 Jul 23;16:6772. doi: 10.1038/s41467-025-62052-x (PMC12287309; doi:10.1038/s41467-025-62052-x)
Supplement: Supplementary file 1 — Supplementary Information [file 41467_2025_62052_MOESM1_ESM.pdf]

Supplementary material for "Estimating excess bound water content due to serpentinisation of mature slow-spreading oceanic crust using Vp/Vs"

Lianjun Li[1]*, Jenny Collier[1]*, Tim Henstock[2], Saskia Goes[1]

[1]*Department of Earth Science and Engineering, Imperial College London, SW7 2BP London, UK.*

[2]*School of Ocean and Earth Science, University of Southampton, SO14 3ZH Southampton, UK.*

*email: l.li21@imperial.ac.uk; jenny.collier@imperial.ac.uk

This document includes:

Tables: Supplementary Table. 1 to Table. 3

Figures: Supplementary Fig. 1 to Fig. 33

Supplementary Table 1 Picking statistics for all observed seismic phases			
Phase	Picking uncertainty (ms)	# of picks	Average pick uncertainty (ms)
Ps	35	16899	35
PbP	25	10735	25
Pg (Pg2 in the upper crust) with DC	30 ms + 0.25 ms/km	19256	32
Pg (Pg2+Pg3) without DC	50 ms + 0.25 ms/km	22166	56
PmP	50/75/100ms based on picking confidence	6932	70
Pn (parts of the Pn phases are not shown in this paper)	50 ms + 0.25 ms/km	33100	74
Sg with DC	150/200/250ms + 0.25 ms/km based on S-wave signal-to-noise-ratio	9272	177
Sg with/without DC		11461	183
SmS		2930	171
Sn		24831	186

Supplementary Table 1. Picking statistics for all observed seismic phases. DC: Downward continuation. Please see Li et al. [1] for details.

Supplementary Table 2 Serpentine bound water content calculation					
% serpentine (β) (1)	Vp (m/s) (2)	Vs (m/s) (3)	Vp/Vs	Poisson's ratio (δ) (4)	H_2O wt %
0	7922	4371	1.81	0.28	0
15	7422	4036	1.84	0.29	2
31	6922	3700	1.87	0.30	4
46	6422	3365	1.91	0.31	6
62	5922	3029	1.95	0.32	8
77	5422	2694	2.01	0.34	10
92	4922	2359	2.09	0.35	12
100	4672	2191	2.13	0.36	13
(1) $\beta = 100\times\frac{w}{13}$ (w is the H_2O wt %, assuming 13 wt % water content for fully serpentinized rocks [2]).					
(2) $Vp = 7299 - 32.5\beta$ from Horen et al.[3].					
(3) $Vs = 4371 - 21.8\beta$ from Horen et al.[3].					
(4) $\delta = \frac{1}{2}[1 - \frac{1}{(Vp/Vs)^2-1}]$ from Christensen [4].					

Supplementary Table 2. Serpentine bound water content calculation. The relationship between serpentinisation (Equation 1) and water content is from Carlson and Miller [2]. The relationship between P- and S-wave velocity and serpentinisation degree (Equation 2 and 3) is from Horen et al. [3]. Poisson's ratio and Vp/Vs ratio (Equation 4) is from Christensen [4].

Supplementary Table 3 Fracture water weight percent calculation

	Fracture water polygon #	Porosity line bounding fracture water polygons (%) φ		Fracture water wt% [1]	Averaged fracture-water wt% assigned for each polygon [2]
Using dunite as the host material in DEM	P1	lower bound	0.0002	0.0062	0.04
		upper bound	0.0025	0.0779	
	P2	lower bound	0.0025	0.0779	0.14
		upper bound	0.0062	0.1938	
	P3	lower bound	0.0062	0.1938	0.45
		upper bound	0.0222	0.7015	
	P4	lower bound	0.0222	0.7015	0.89
		upper bound	0.0337	1.0736	
	P5	lower bound	0.0337	1.0736	1.46
		upper bound	0.0569	1.8428	
	P6	lower bound	0.0569	1.8428	2.25
		upper bound	0.0808	2.6625	
	P7	lower bound	0.0808	2.6625	3.46
		upper bound	0.1251	4.2599	
	P8	lower bound	0.1251	4.2599	5.42
		upper bound	0.1848	6.5894	
Using gabbro as the host material in DEM	P1	lower bound	0.0002	0.0069	0.03
		upper bound	0.0017	0.0591	
	P2	lower bound	0.0017	0.0591	0.10
		upper bound	0.0041	0.1427	
	P3	lower bound	0.0041	0.1427	0.34
		upper bound	0.0154	0.5399	
	P4	lower bound	0.0154	0.5399	0.69
		upper bound	0.024	0.8461	
	P5	lower bound	0.024	0.8461	1.20
		upper bound	0.0435	1.5537	
	P6	lower bound	0.0435	1.5537	1.94
		upper bound	0.0643	2.3292	
	P7	lower bound	0.0643	2.3292	3.11
		upper bound	0.1046	3.8961	
	P8	lower bound	0.1046	3.8961	5.08
		upper bound	0.1614	6.2610	

(1) For the dunite case, wt%=100%× $\frac{1.03\varphi}{3.31(1-\varphi)+1.03\varphi}$, φ is the porosity, water density=1.03 g/cm^3, dunite matrix density=3.31 g/cm^3;

For the gabbro case, wt%=100%× $\frac{1.03\varphi}{2.968(1-\varphi)+1.03\varphi}$, φ is the porosity, water density=1.03 g/cm^3, gabbro matrix density=2.968 g/cm^3.

(2) Average of the fracture water wt% calculated for the lower and upper porosity bounds

Supplementary Table 3. Fracture water weight percent calculation. The fracture water polygons, numbered P1 to P8 and bounded by neighbouring porosity lines, are shown in Fig. 6b and Supplementary Fig. 31b, c. Velocity points with Vp/Vs > 1.9 which fall into each fracture water polygon are assigned an averaged fracture-water weight percent (wt%) between the fracture-water weight percent (wt%) calculated from the lower and upper porosity bounds defining these polygons.

Supplementary Fig. 1

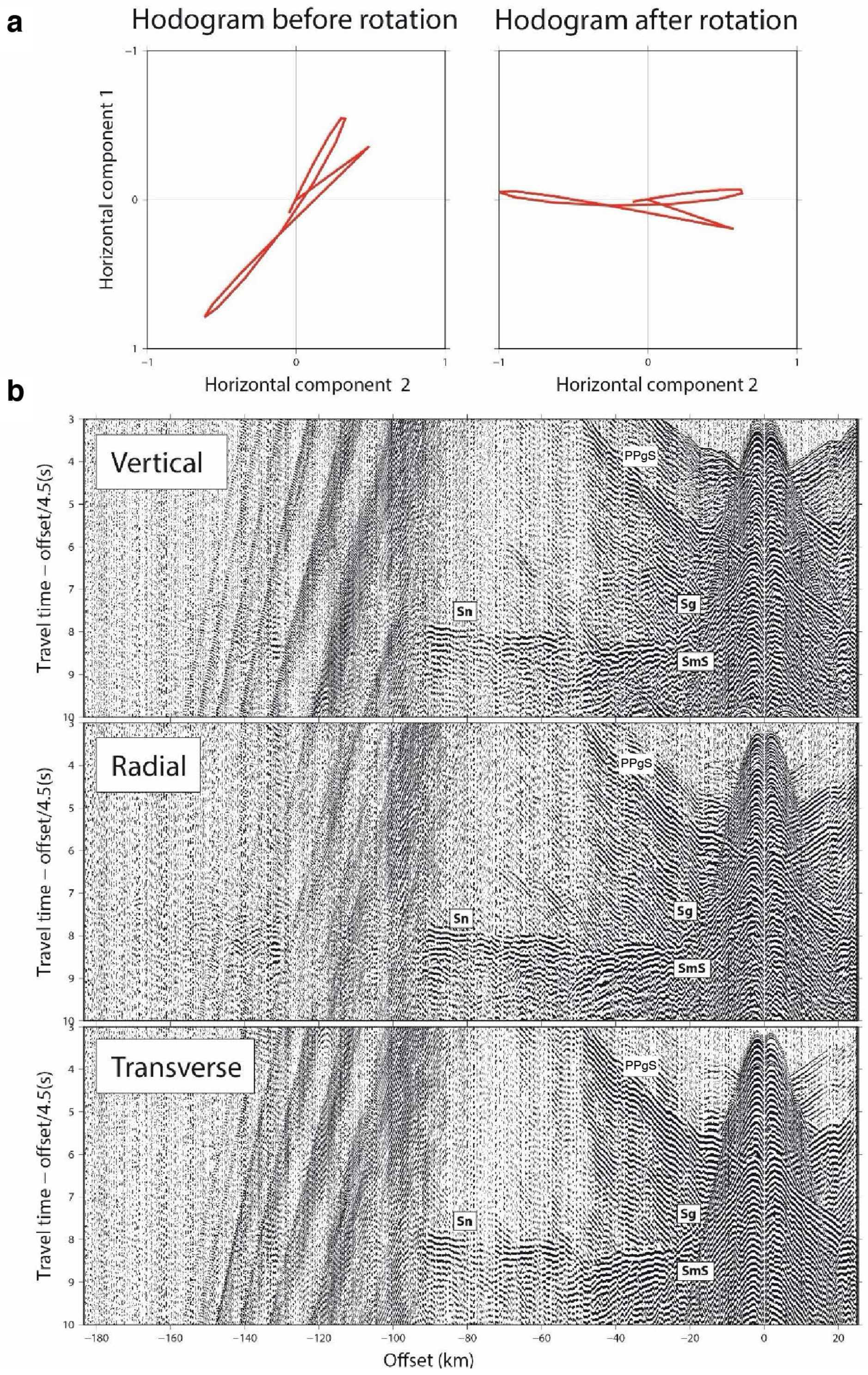

Supplementary Fig. 1. Hodogram analysis. **a** Hodograms before and after the rotation of the two horizontal geophones to form the radial and transverse components. **b** Seismograms of the vertical, radial, and transverse components after rotation, reduced by a velocity of 4.5 km/s. See Supplementary Fig. 2 for the S-wave phase name.

Supplementary Fig. 2

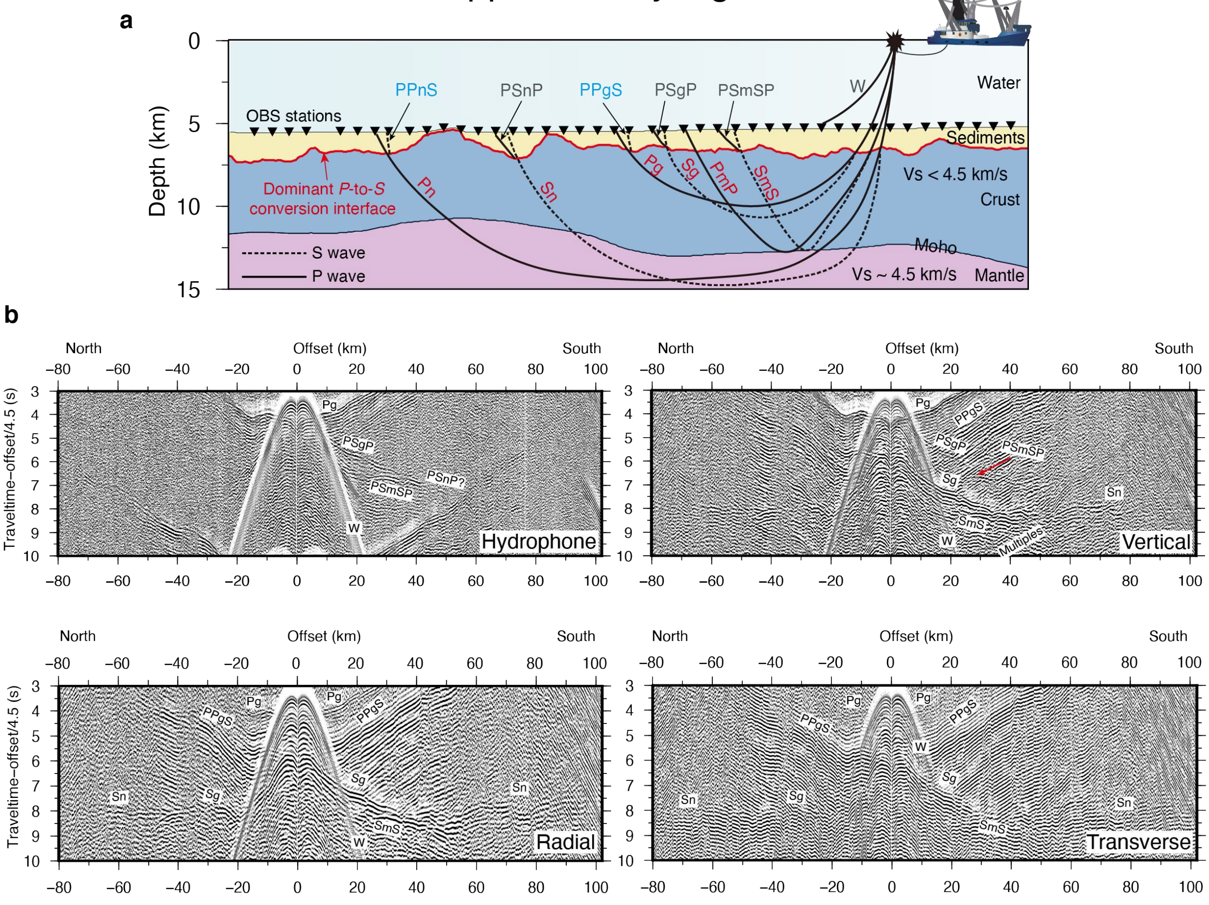

Supplementary Fig. 2. Identifying P-to-S and S-to-P converted phases on OBS record. a Cartoon illustrating the main observed S-wave arrivals with P-to-S and S-to-P conversions at the sediment-basement interface. Phases used in tomography are in red, including Pg, PmP, Pn, Sg, SmS and Sn. Phases used in time correction for S-wave modelling are in blue, including PPgS and PPnS. Phases not used in the analysis are in grey, including PSgP, PSmSP and PSnP. **b** Comparison of main phases on the four available components (hydrophone, vertical, radial and transverse). Note by comparison among the 4 components the picked S-waves (Sg/Sn/SmS phases) that converted from source P-wave at the sediment-basement interface during the downward propagation leg are clear on the 3 geophone components. The double-mode converted phases PSgP, PSnP, PSmSP are only intermittently seen on hydrophone and vertical components with relatively weaker amplitudes and therefore are not used in analysis. W—water wave; Pg—crustal refractions; PmP—Moho reflections; Pn—mantle refractions. For converted S-waves, phases that are converted to S-waves on the way down are given 2 or 3 letter codes, and those that are converted on the way up or on both the way down and up are given 4 or more letter codes.

Supplementary Fig. 3

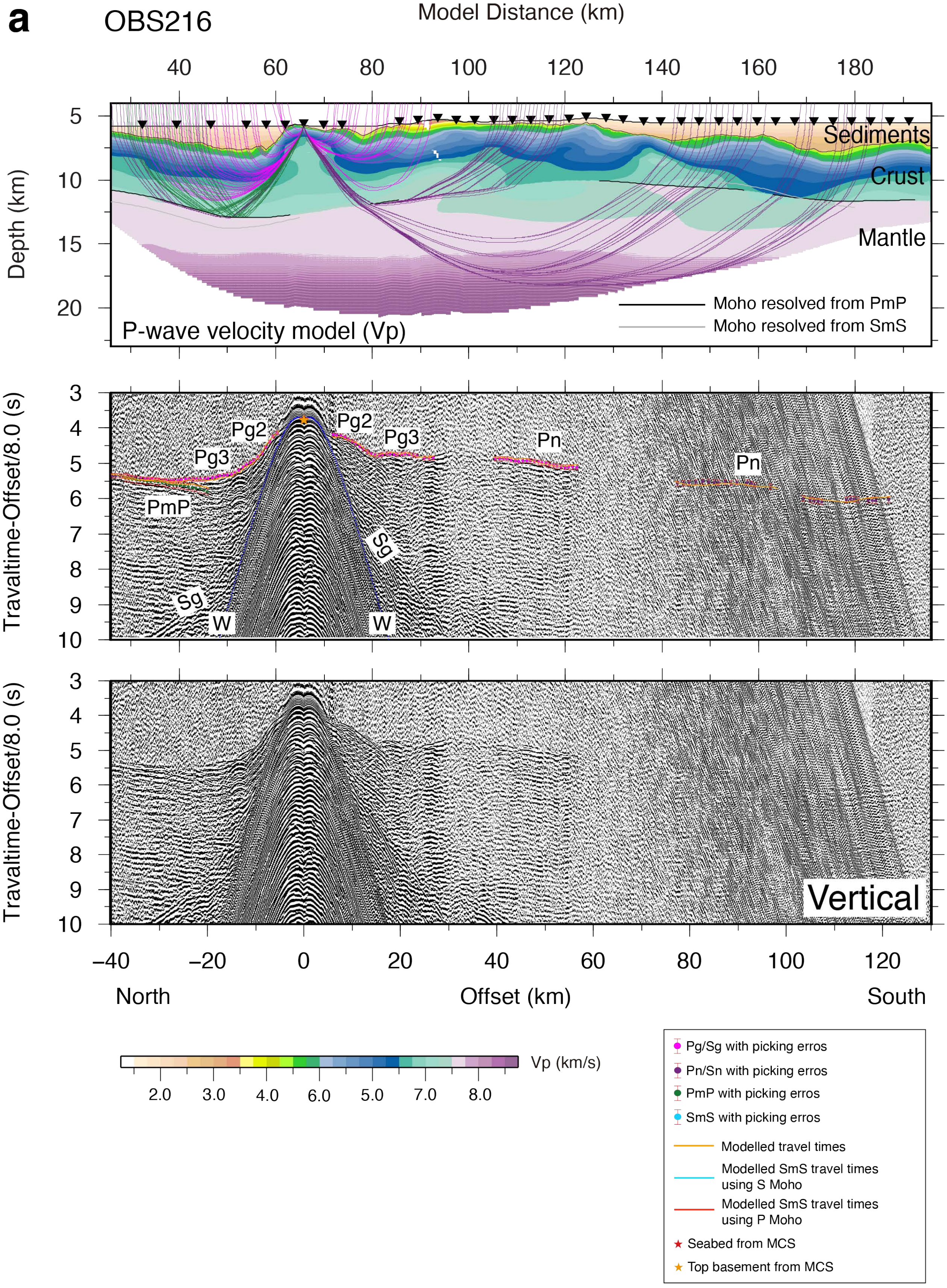

Supplementary Fig. 3

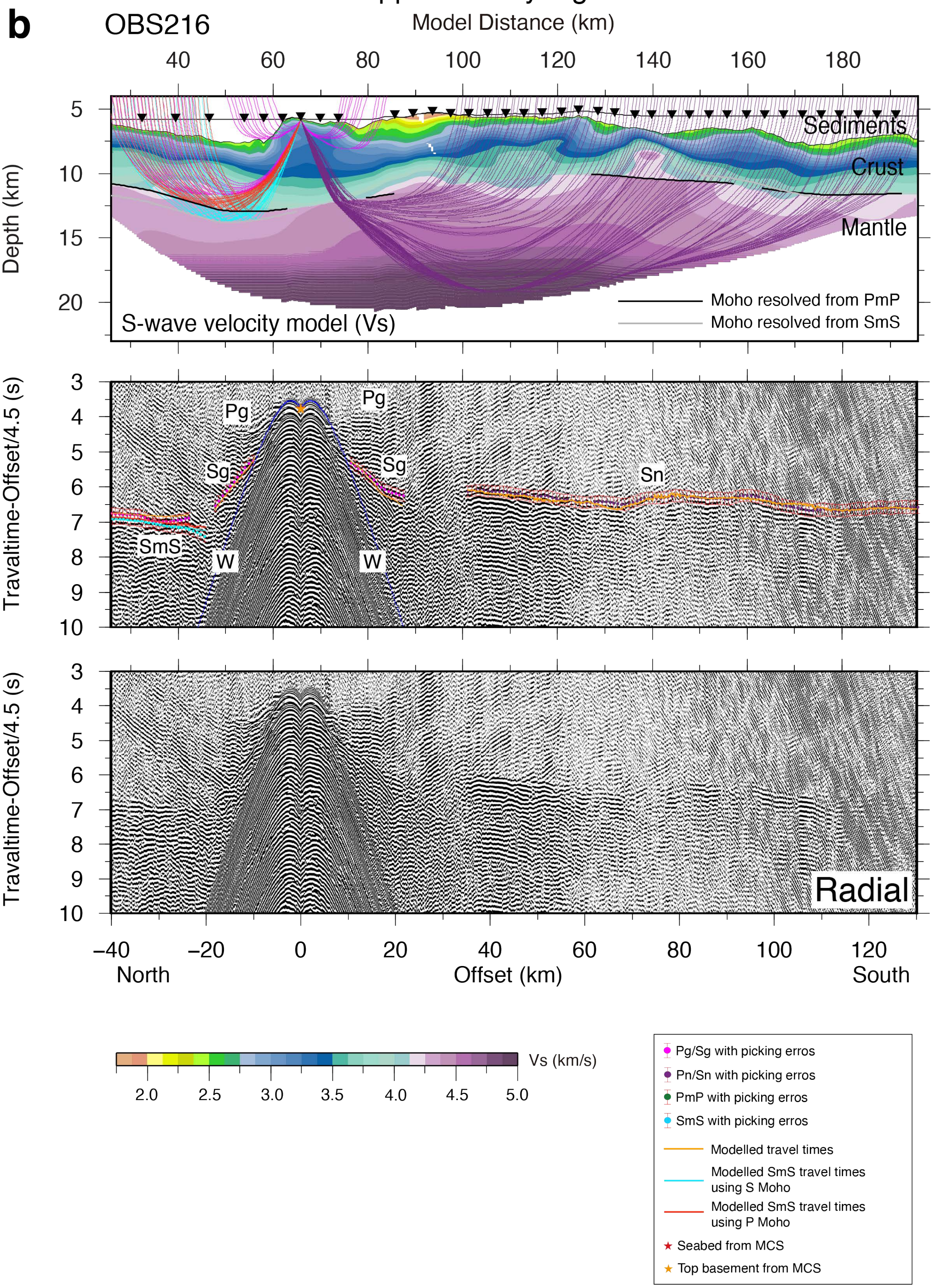

Supplementary Fig. 3

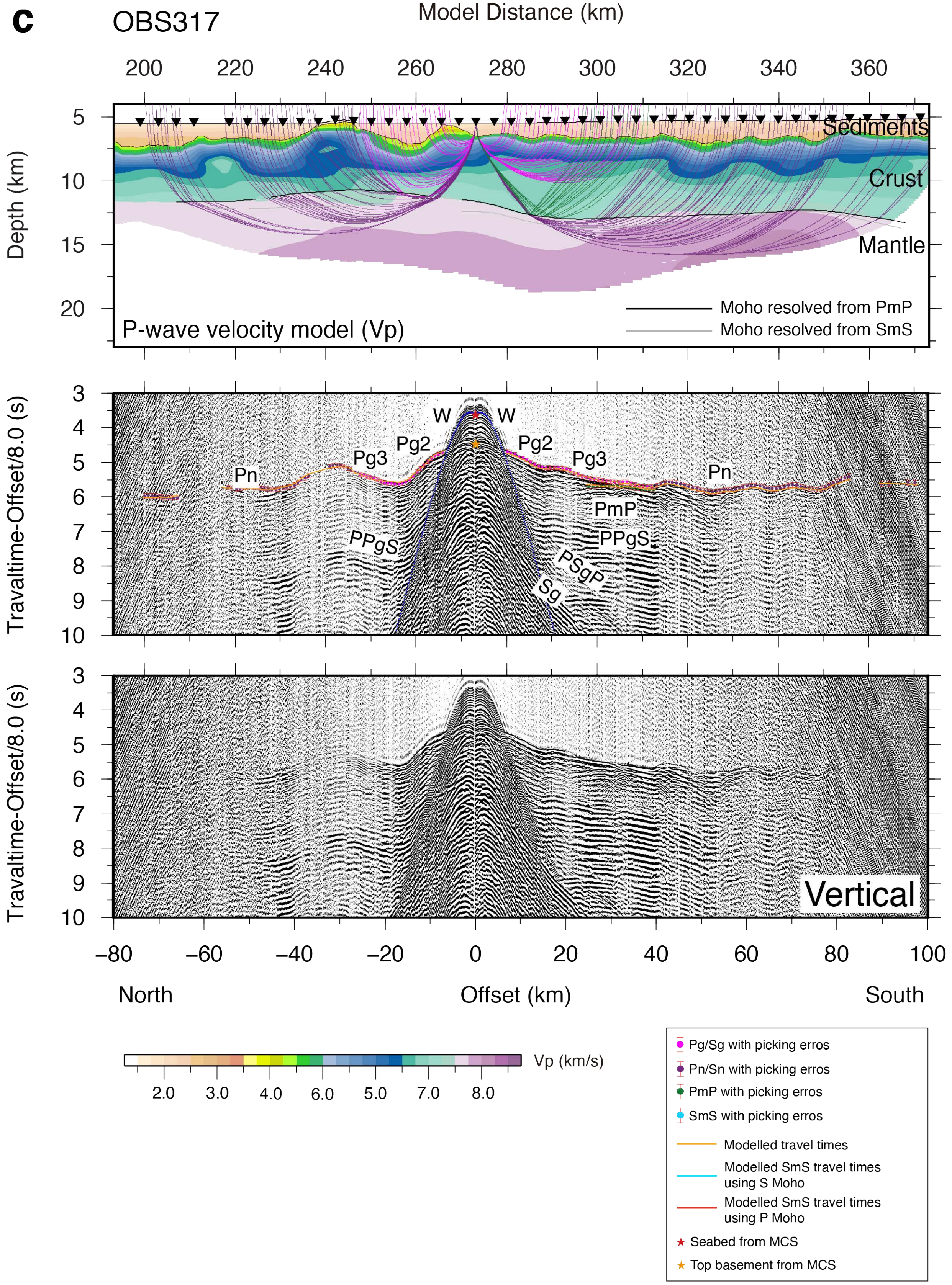

Supplementary Fig. 3

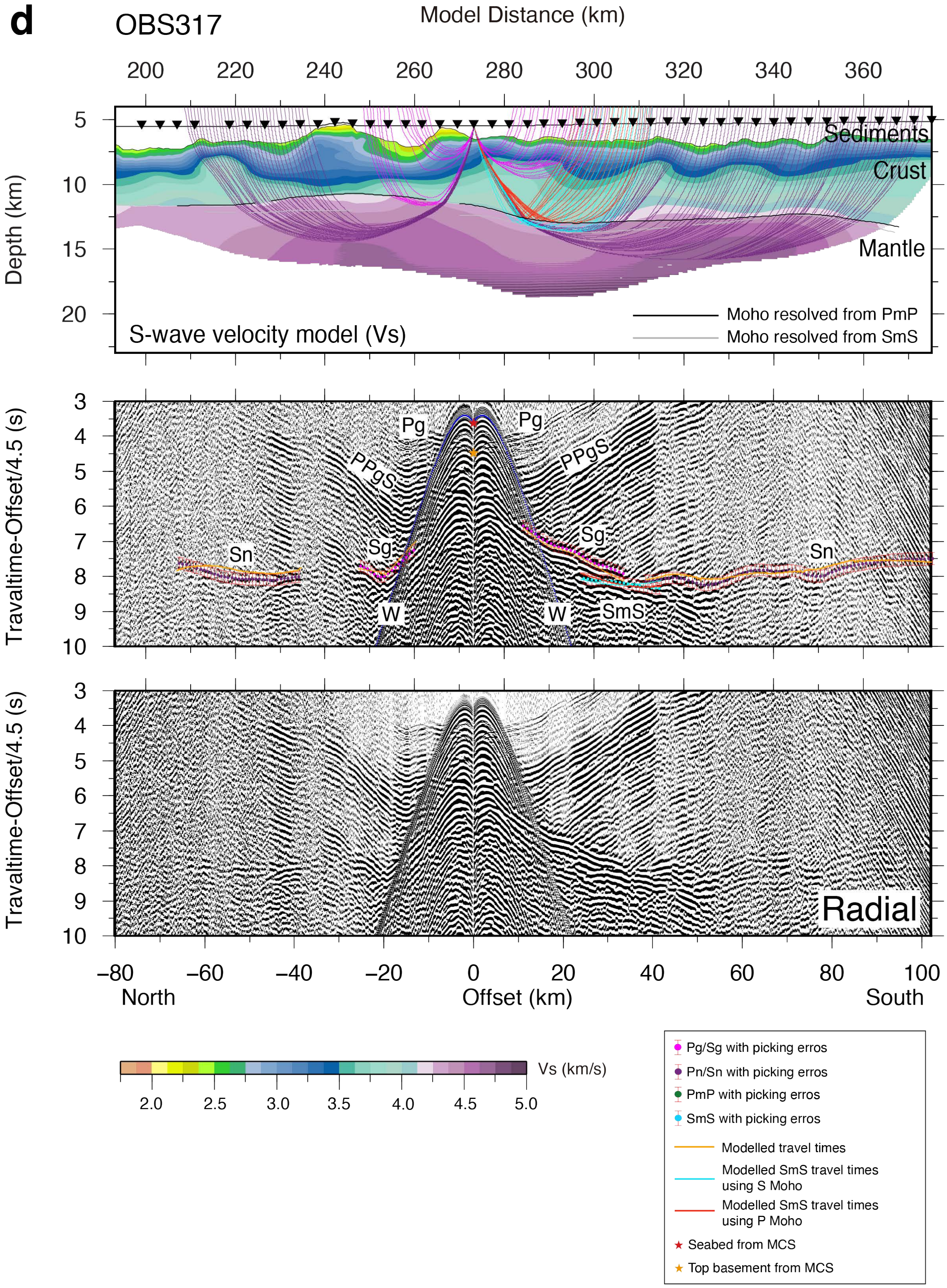

Supplementary Fig. 3. Summary of tomographic inversion of P- and S-wave phases at OBS 216 (a, b) and OBS 317 (c, d), respectively. The upper panels show the ray diagram, and the lower two panels show the OBS data with (middle) and without (bottom) the observed

(coloured dots with picking uncertainties) and modelled (orange solid lines) arrivals. Pg2—upper crustal refractions; Pg3—lower crustal refractions; other phases see Supplementary Fig. 2. All the models are masked by ray coverage of S-wave data (see Supplementary Fig. 14). MCS—multichannel seismic data. The Moho depth was inverted using PmP phases (P Moho: black solid lines) and fixed during S-wave inversion. The Moho depth inverted using SmS phases (S Moho: grey solid lines) is used for comparison with the P Moho (see "Methods" section). Unless specified, P Moho and S Moho are plotted in subsequent figures with the same colour code.

Supplementary Fig. 4

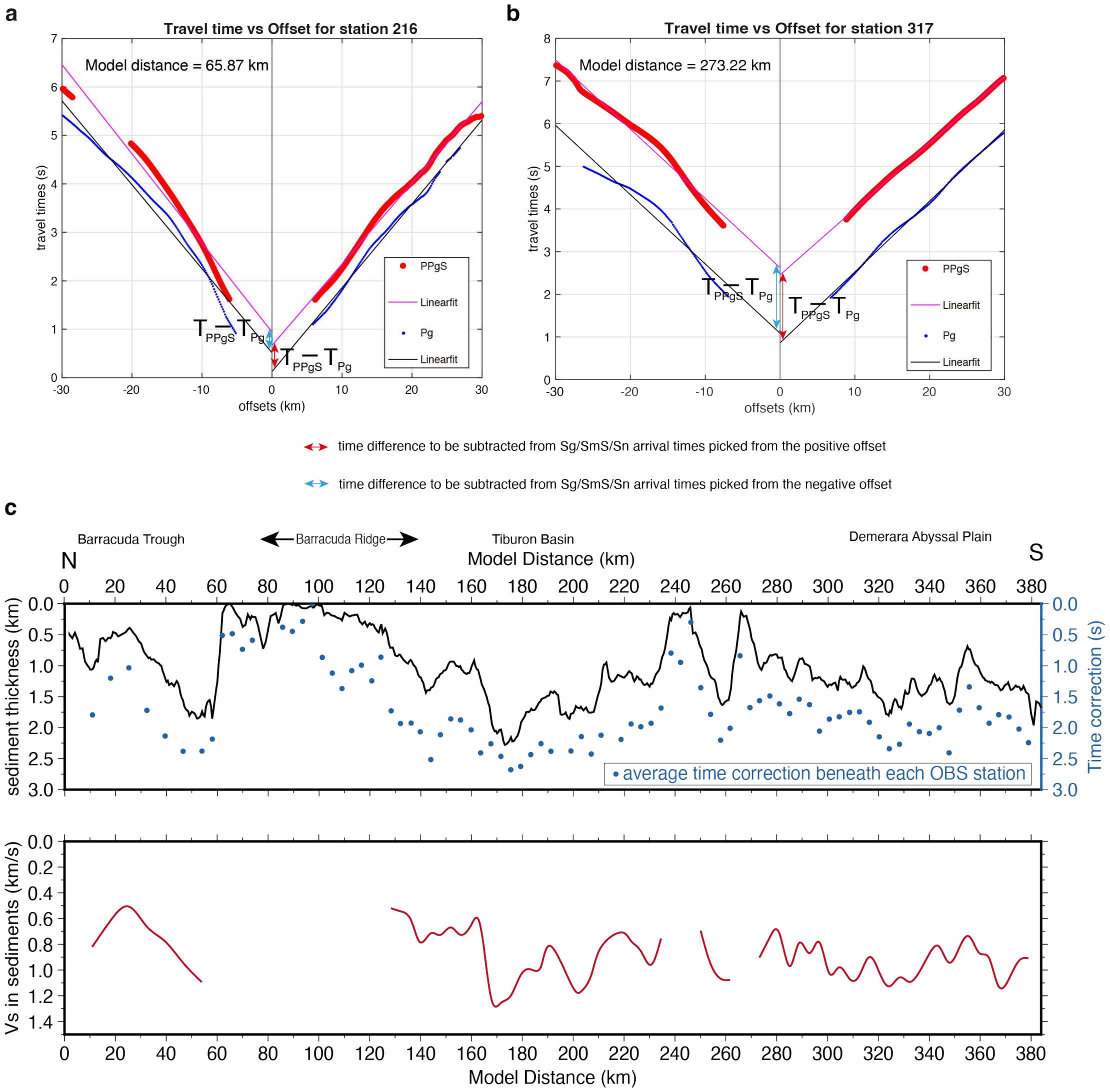

Supplementary Fig. 4. Time correction for S-wave modelling in TOMO2D for OBS 216 (a) and OBS 317 (b). The time difference (absolute values) applied in the correction is the difference (red and blue double arrows) between the intercept time of the linear fitting of picked PPgS (if present) and Pg arrivals. The values calculated from the positive and negative offset branches were averaged. This difference was then subtracted from Sg/SmS/Sn arrival times to produce "effective PSP arrivals", i.e., converted S-wave propagating in the sub-basement but P-wave in sediments. In this way, the symmetry of the ray path is given to the converted phases to be used in TOMO2D [5], and the sediment layer is assigned P-wave velocities throughout the modelling. **c** Average time correction for each OBS station (blue dots). Also shown is sediment thickness (solid black line), and S-wave velocity (Vs) estimation (solid red line). Sediment thickness is defined as the distance between the seabed and the sediment-basement interface measured from coincident MCS. Vs in sediments is the sediment thickness divided by the difference between time correction and the P-wave travel time in the sediments beneath each OBS station.

Supplementary Fig. 5

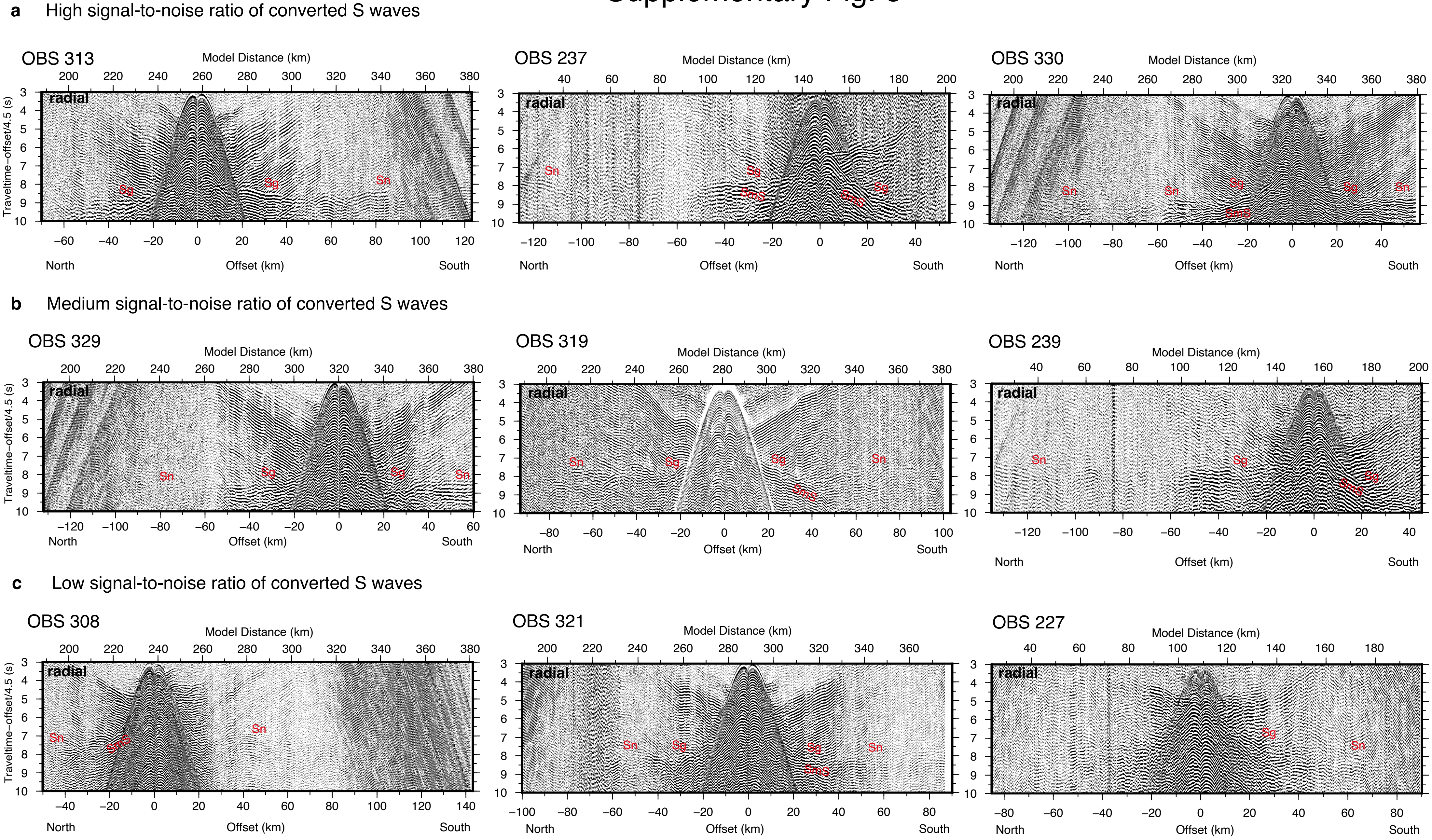

Supplementary Fig. 5. **Examples of S-wave data quality**. Selected OBS records showing high (**a**), medium (**b**) and low (**c**) signal-to-noise ratio (SNR) for converted S-wave arrivals. The classification is mainly based on the S-wave amplitude, consistency and coherence. A starting picking uncertainty of 0.75T, 1.0T and 1.25T (where T=200 ms which is an average period of S-waves for all offset ranges) was assigned to picked travel time S-wave arrivals. For S-wave phases see Supplementary Fig. 2.

Supplementary Fig. 6

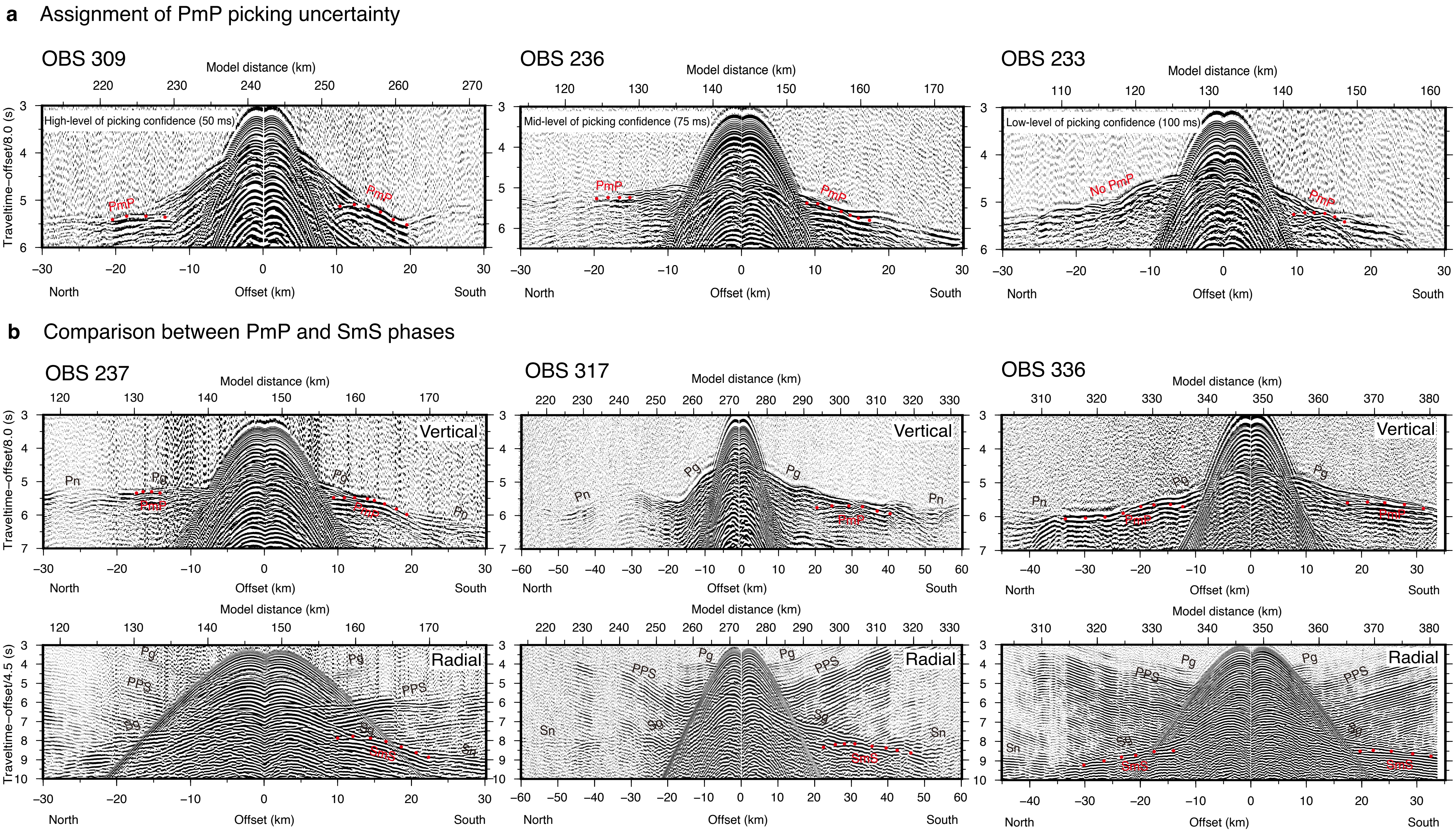

Supplementary Fig. 6. PmP and SmS phases on OBS records. **a** Contrasting levels of picking confidence for PmP phases on three example OBS records. Corresponding PmP arrivals were assigned with 50 ms, 75 ms and 100 ms picking uncertainty based on the consistency, coherence, and amplitude of the PmP reflector (red dots). **b** Comparison between PmP and SmS phases on vertical (upper panel) and radial (lower panel) components, respectively. PmP and SmS phases present at comparable offset ranges.

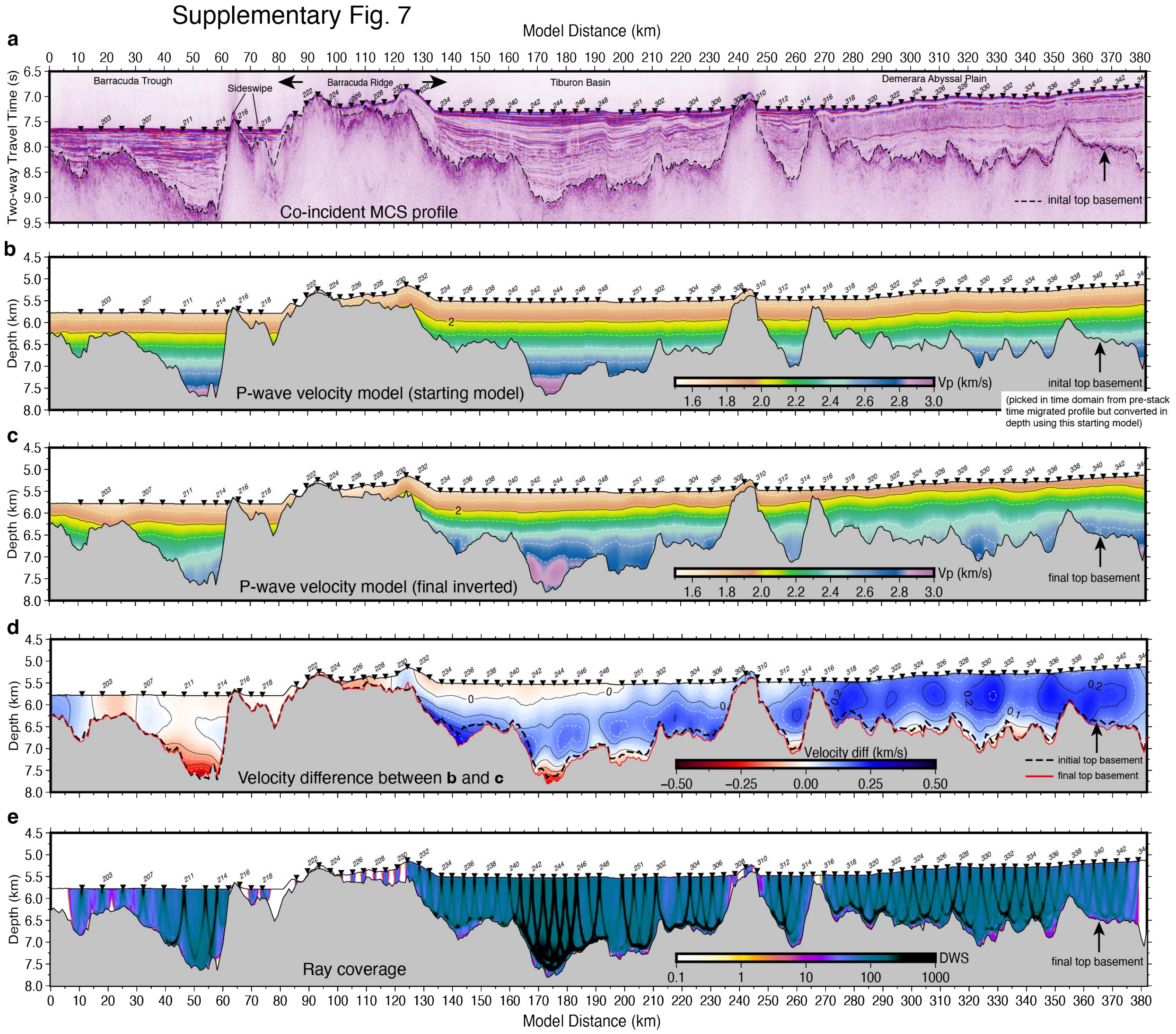

Supplementary Fig. 7. Tomographic inversion of the sedimentary P-wave model. Southern part of this model is presented in Li et al.[1] and the full model is included here for completeness. The details for inverting sedimentary model and the top basement are present in Li et al. [1]. **a** Co-incident pre-stack time migrated multi-channel seismic (MCS) profile. The top basement horizon was picked and then converted to depth using sedimentary P-wave starting model shown in b. **b** Starting model with a single seabed velocity of 1.7 km/s and vertical velocity gradient of 0.65 /s [1]. **c** Final inverted sedimentary model. **d** Difference between starting and final models. **e** Derivative weight sum (DWS) of the final model representing ray coverage. Note the velocity gradient change in c from south to north suggesting reduced terrigenous input from the Orinoco delta. The P-wave velocity (Vp) model displays a sediment cover of variable thickness that includes the Cenozoic sequences traced back to the early Eocene [6]. The thickest sediment layer is found under Tiburon Basin and Barracuda Trough (> 2.5 km) with Vp increased from ~1.7 to 2.8 km/s. In Demerara Abyssal Plain (DAP) at the south of the line, ~ 1.5 to 2 km thick sediments with Vp

increasing from ~ 1.9 km/s to 2.6 km/s. The difference in sedimentary velocity-depth profiles extracted from the south and north corresponds well to the fact that more terrigenous materials from the Orinoco delta are deposited in the southern part of the line compared to the north.

Supplementary Fig. 8

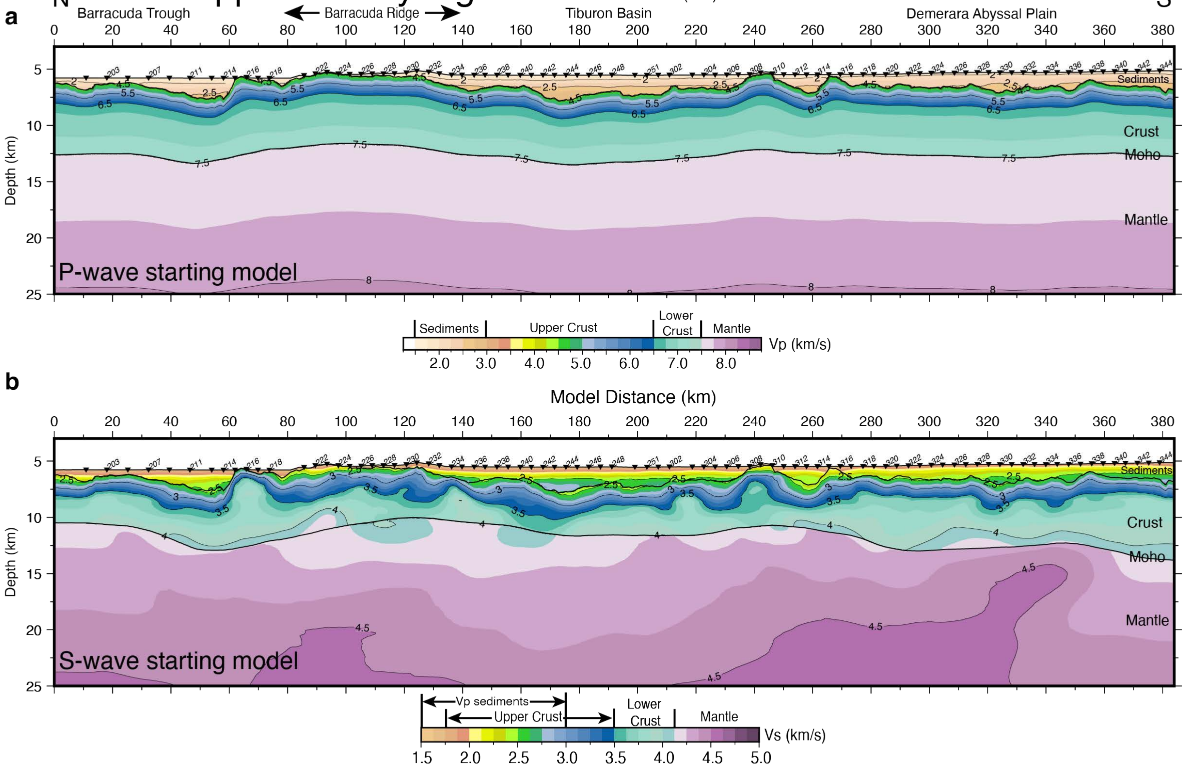

Supplementary Fig. 8. **Starting models for P- (a) and S-wave (b) travel-time tomographic inversions in the crust and mantle.** The final inverted P-wave sedimentary model included in (**a**) and (**b**) was discussed in Li et al.[1] and shown in Supplementary Fig. 7. P-wave velocity (Vp) starting model was guided by global OBS compilations of velocity-depth profiles for the slow-spreading old oceanic crust (> 7.5 Ma) from Christeson et al.[7]. The top upper crustal velocity is 4.5 km/s in (**a**), which is estimated from the tomographic inversion result for the southern portion of the seismic line from Li et al.[1]. S-wave velocity (Vs) starting model in (**b**) was converted from the final inverted P-wave model using Vp/Vs=1.8 in the crust and mantle.

Supplementary Fig. 9

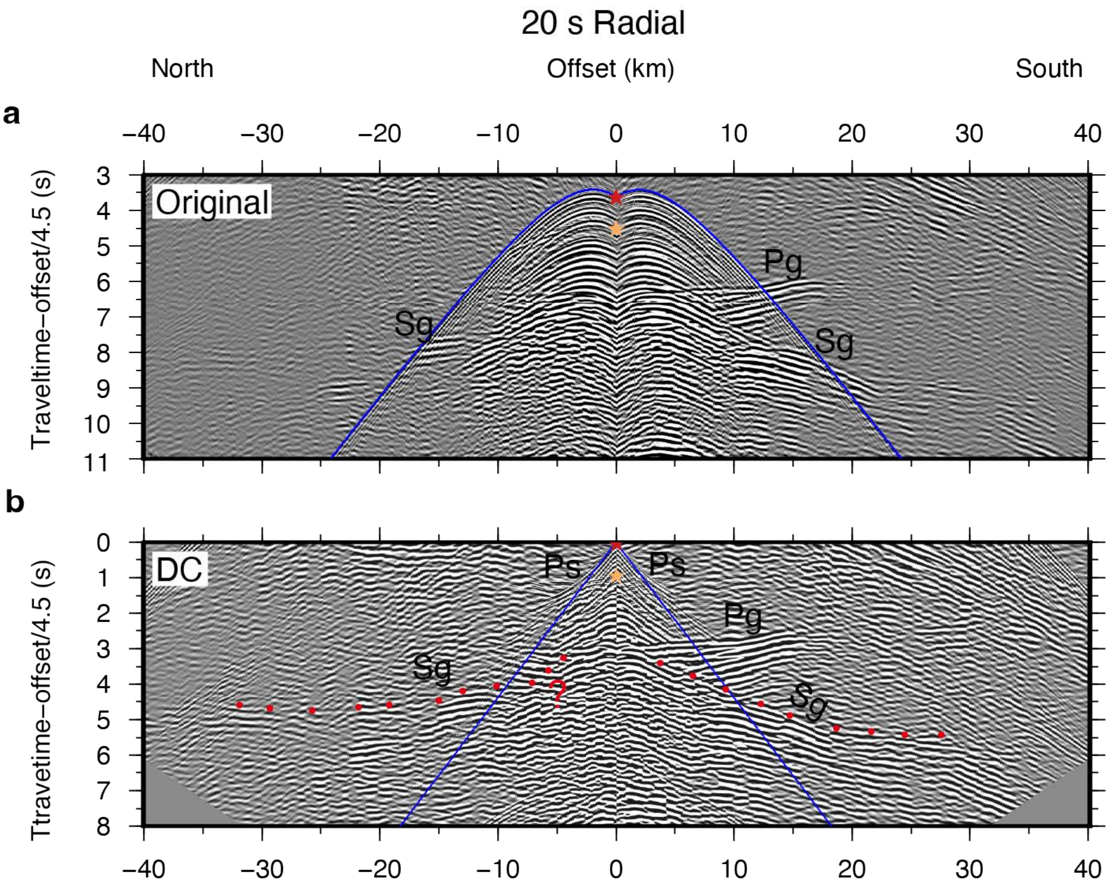

Supplementary Fig. 9. A 20 s shot interval S-wave record in the radial direction before (a) and after (b) the application of DC. Note this process is not as essential as for P-wave modelling [1] because on horizontal components the water wave (blue line) is weaker and thus causes less interference with the S-wave picking. Red star—seabed picked from MCS; orange star—top basement picked from MCS.

Supplementary Fig. 10

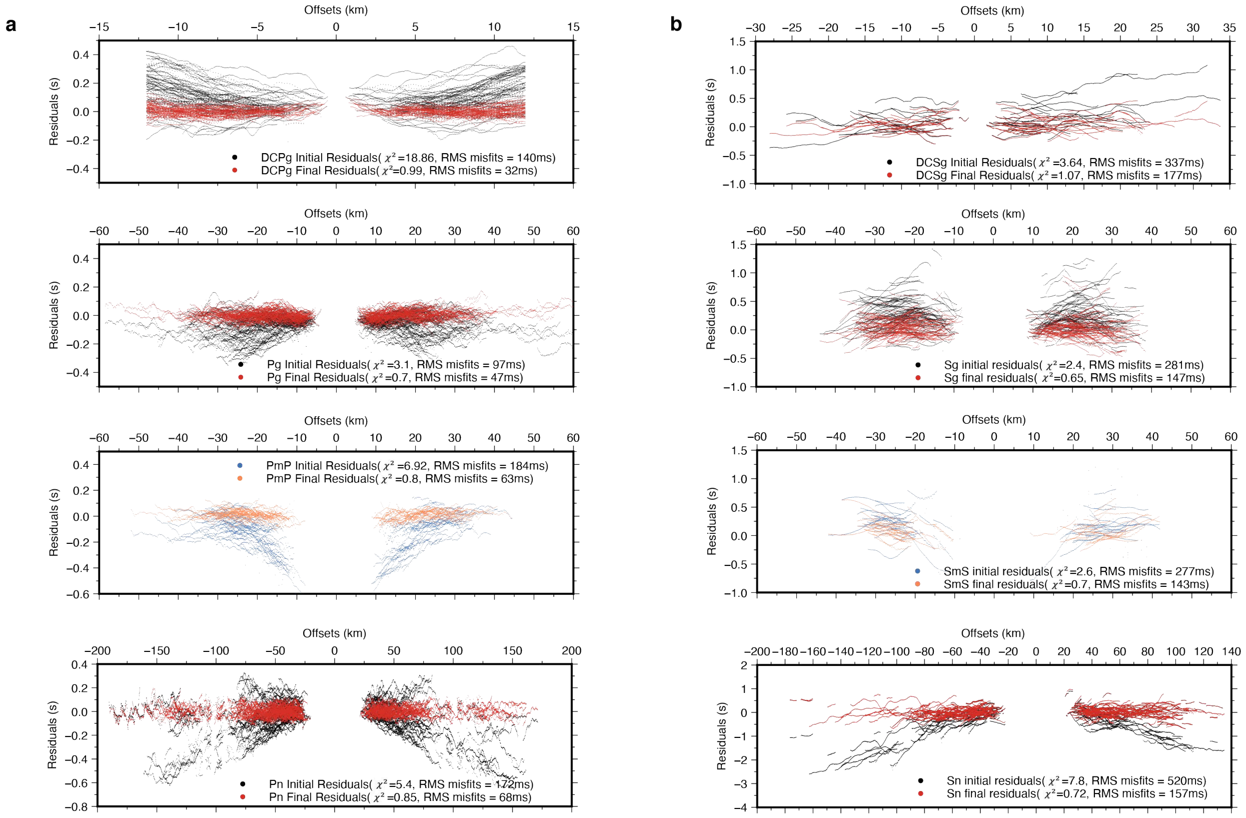

Supplementary Fig. 10. Summary plots for travel time residuals of all picked phases. Travel time residuals (RMS misfits) between the picked and predicted travel times for all P-wave (**a**) and S-wave (**b**) phases as a function of the source-receiver offsets in the starting/initial model (black and blue dots) and the final tomographic model (red and orange dots). P- and S-wave phases are labelled in each figure. Note the similarity in performance of the S-wave inversion to the P-wave. After inversion, travel time residuals of all the phases were reduced with χ^2 approaching to 1, suggesting the final model fits the data adequately.

Supplementary Fig. 11

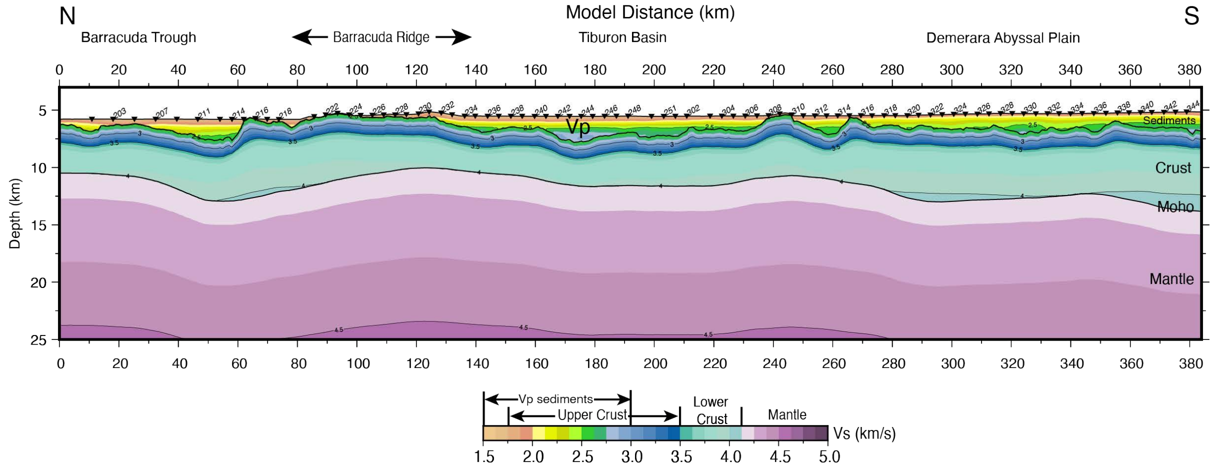

Supplementary Fig. 11. A starting model for a comparison test of S-wave velocity modelling. The model was converted from the initial P-wave crustal and mantle velocities shown in Supplementary Fig. 8a with a Vp/Vs ratio of 1.8. The results shown in Supplementary Figs. 12 and 13 were generated from this simplified S-wave starting model and the S-wave starting model with Vp prior information shown in Supplementary Fig. 8b.

Supplementary Fig. 12

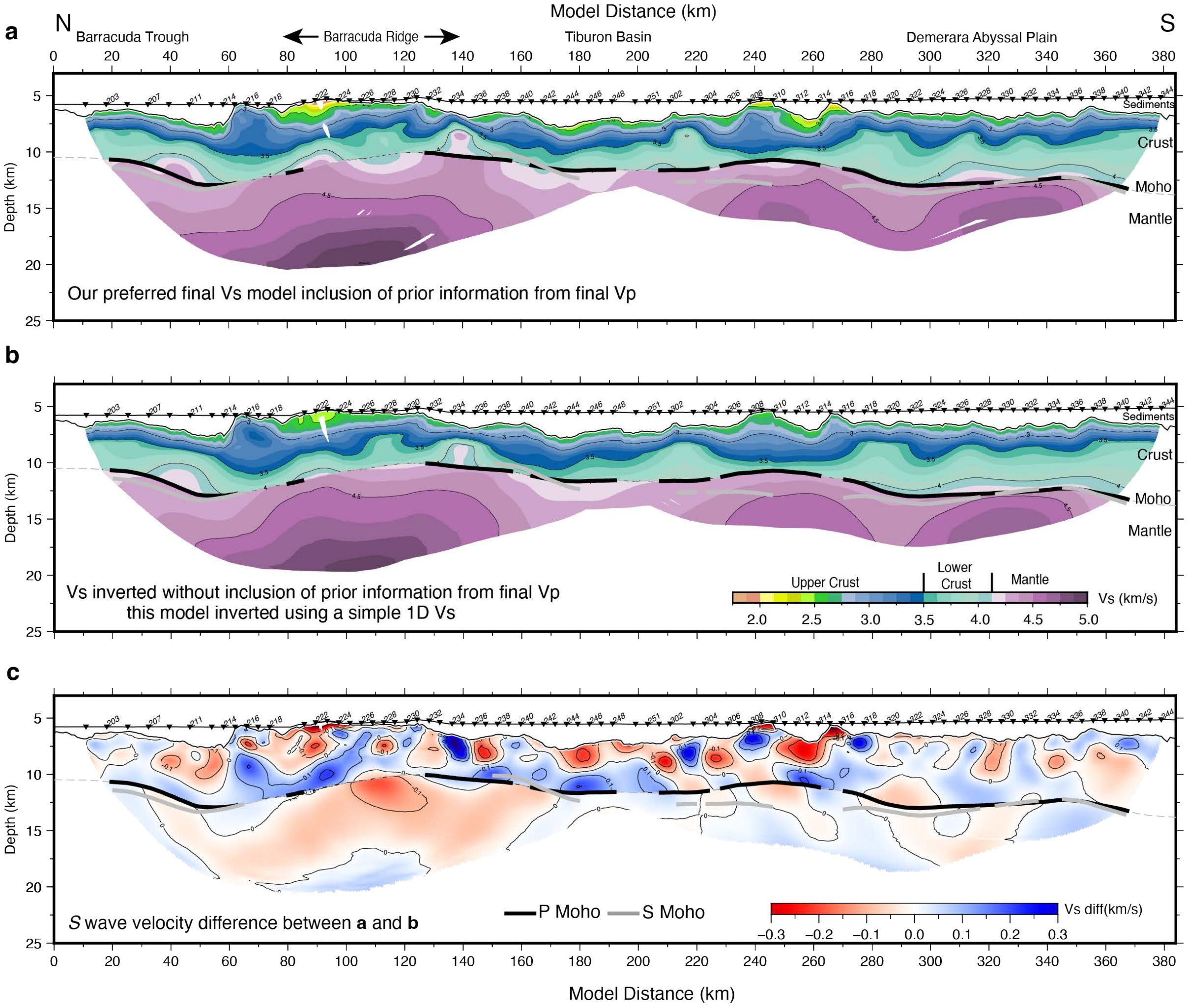

Supplementary Fig. 12. Final S-wave velocity models using different starting models. a Our preferred final S-wave velocity model generated from a starting model with a prior structure shown in Supplementary Fig. 8b. **b** The final S-wave model generated from a simplified starting model shown in Supplementary Fig. 11. Note high similarity between the two models except for localised differences at topographic highs due to the effect of inclusion of prior P-wave velocity information on the final S-wave inversion results. **c** The difference between a and b. Note the small difference generally below ± 0.1 km/s throughout the model, suggesting that the final results depend less on the starting model.

Supplementary Fig. 13

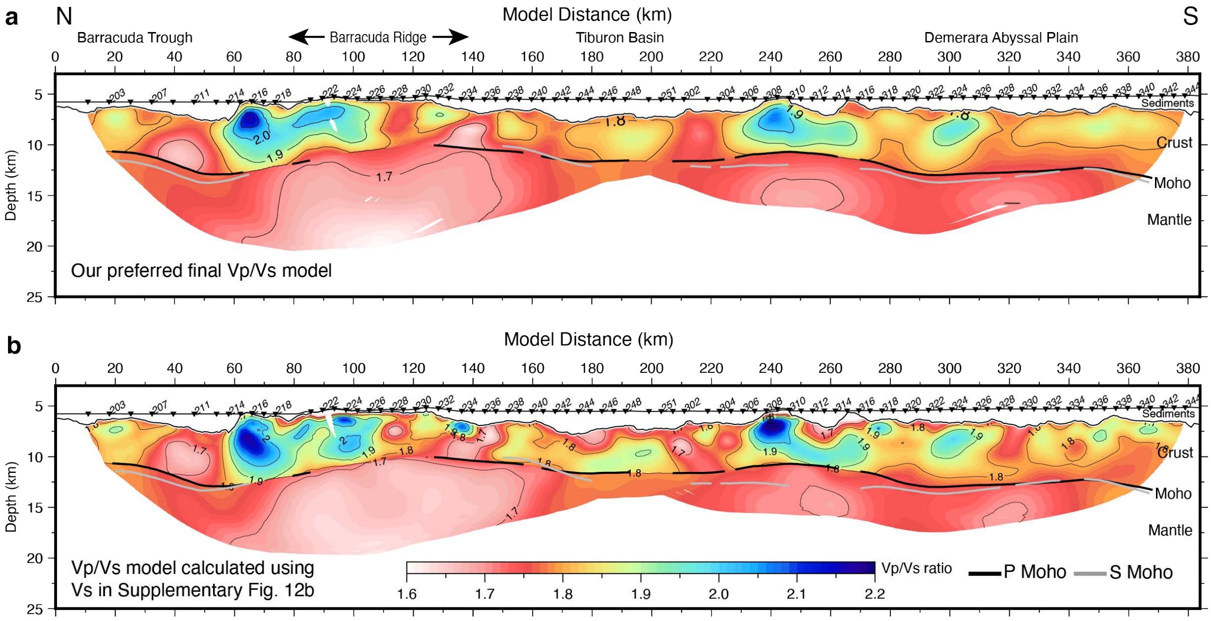

Supplementary Fig. 13. Final Vp/Vs model comparison. a Our preferred Vp/Vs model using the S-wave velocity model from Supplementary Fig. 12a. **b** The final Vp/Vs model using the S-wave velocity model from Supplementary Fig. 12b.

Supplementary Fig. 14

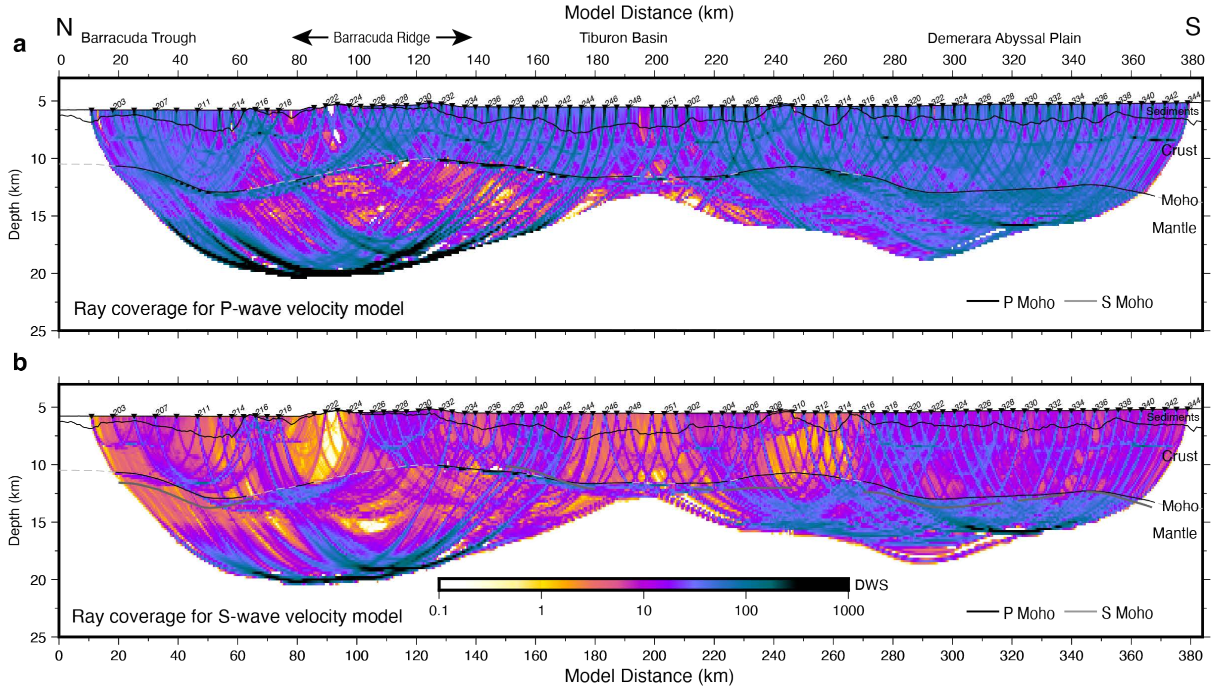

Supplementary Fig. 14. Ray coverage. Derivative Weight Sum (DWS) plot of the final P- (**a**) and S-wave (**b**) models representing ray coverage. Note the much higher ray coverage of P-wave velocity model compared to the S-wave velocity model.

Supplementary Fig. 15

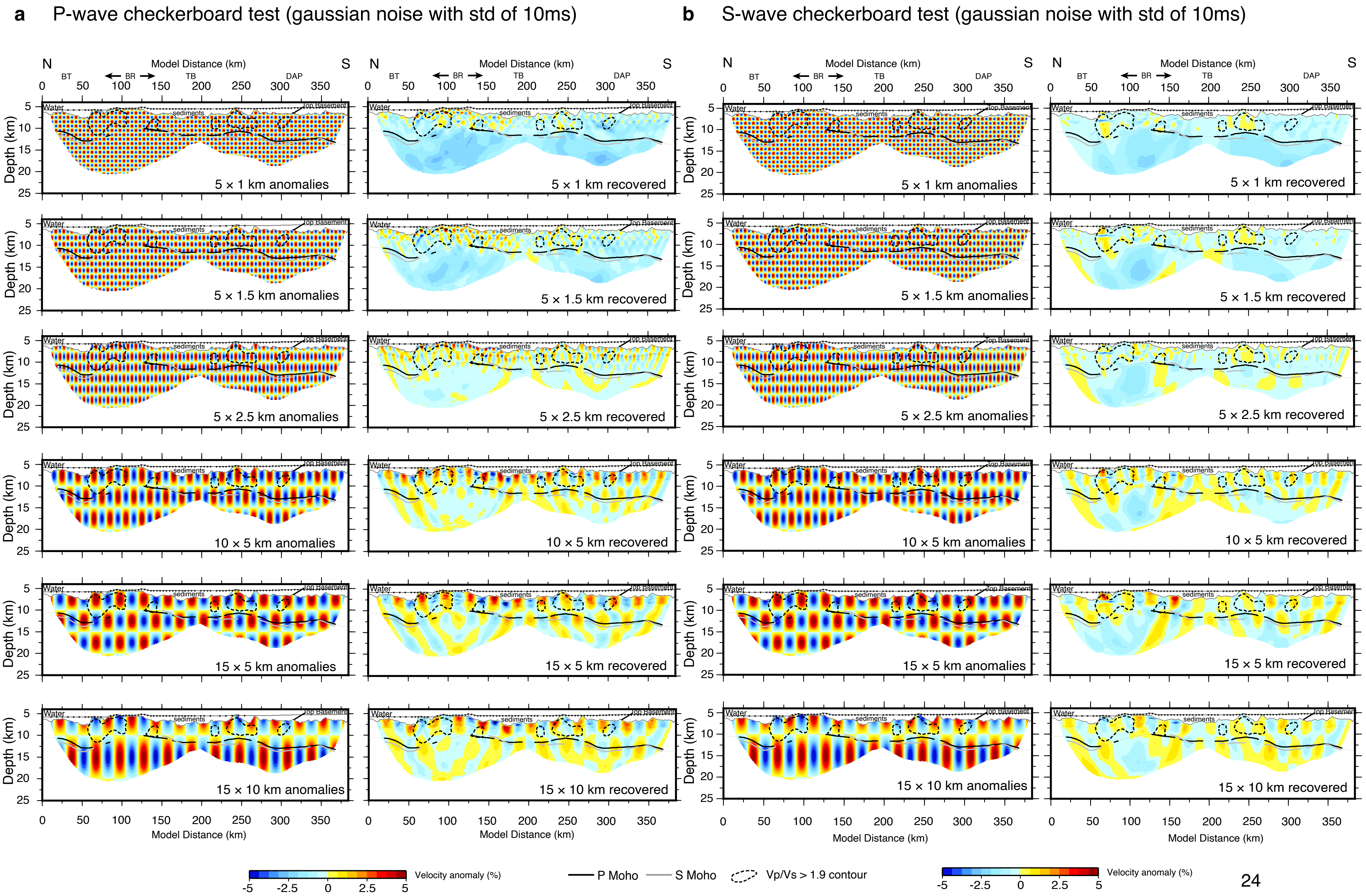

Supplementary Fig. 15

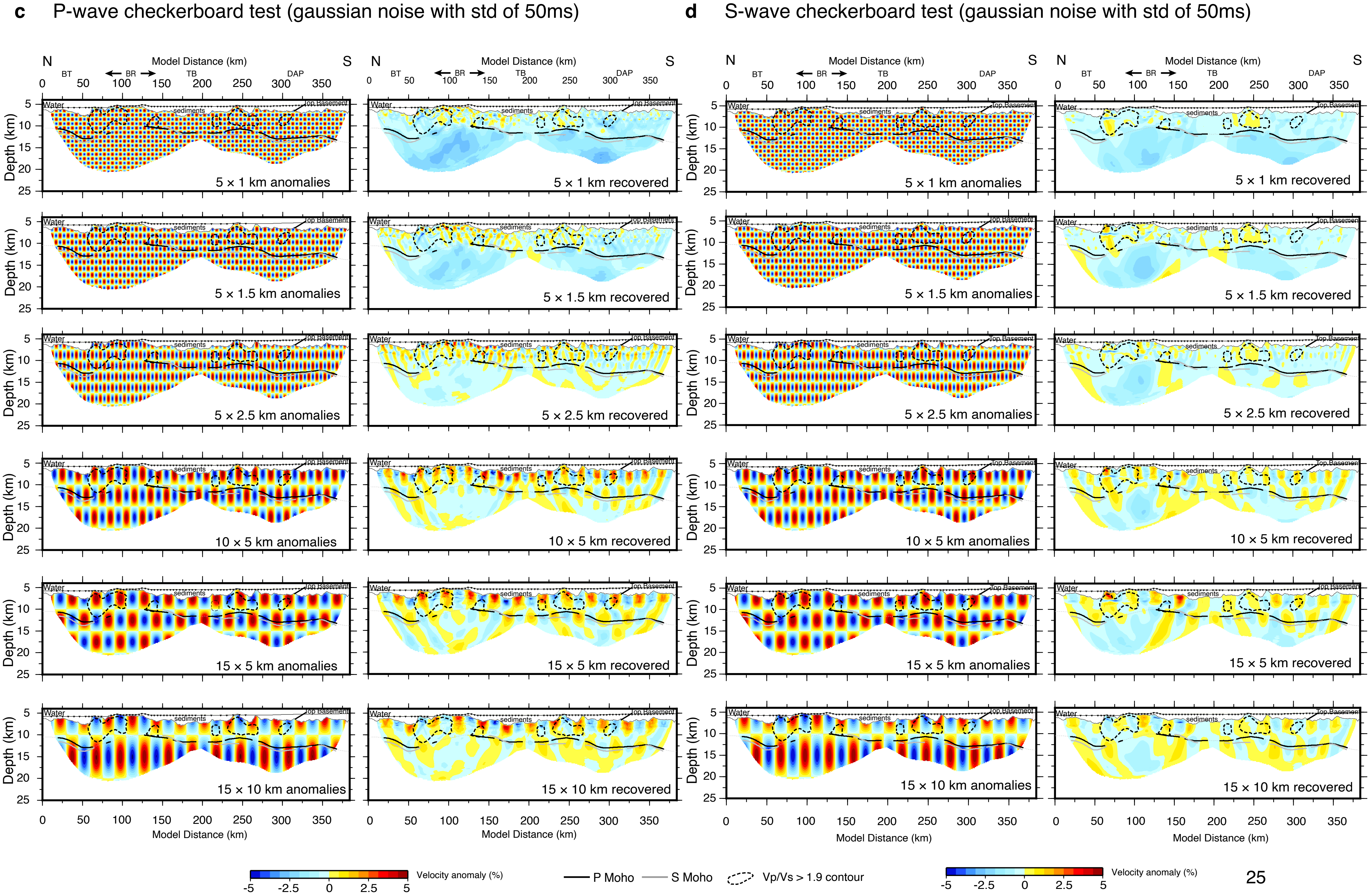

Supplementary Fig. 15

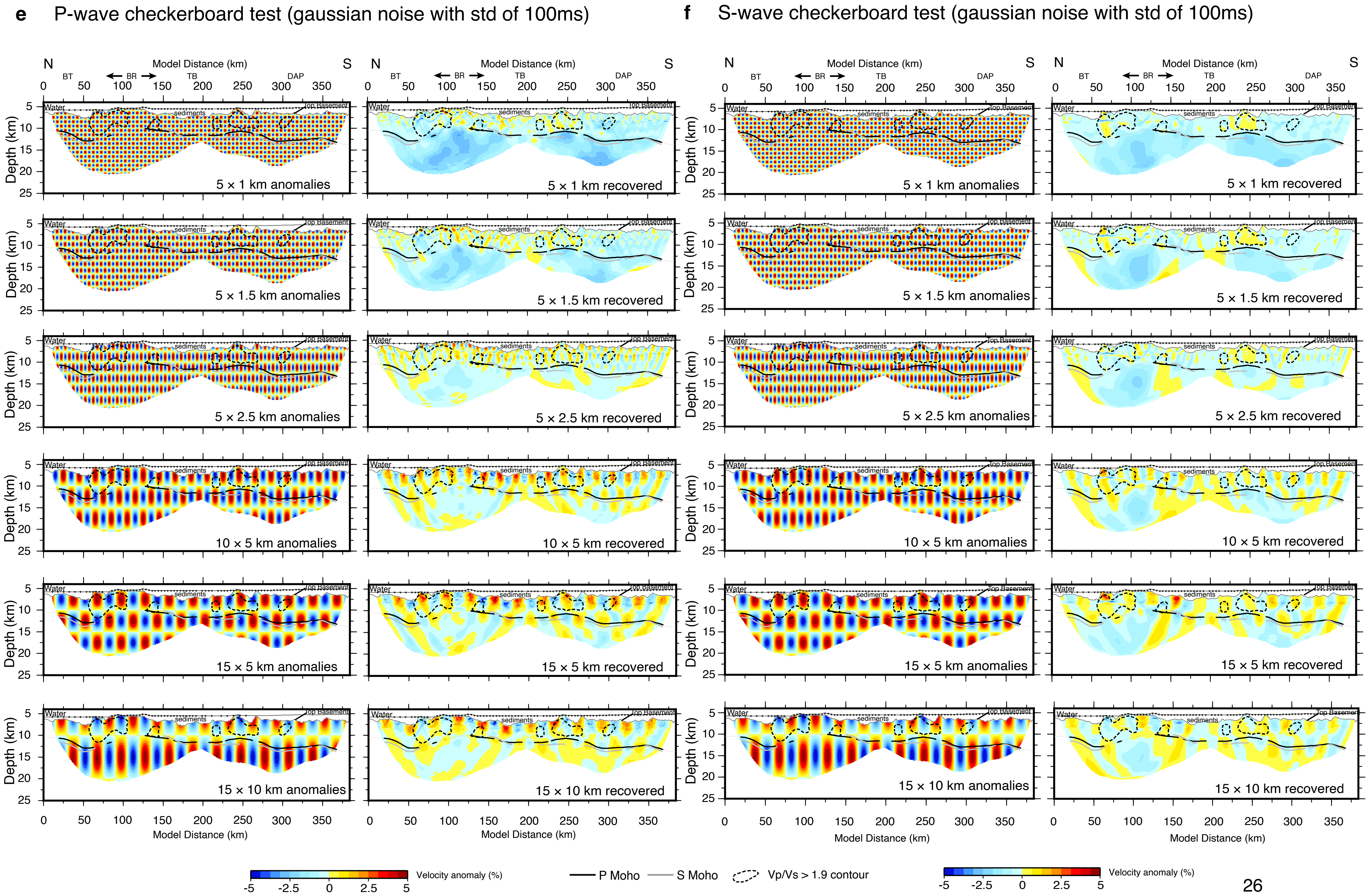

Supplementary Fig. 15. Checkerboard tests. Checkerboard tests at a scale of 5 × 1 km, 5 × 1.5 km, 5 × 2.5 km, 10 × 5 km, 15 × 5 km and 15 × 10 km for final P- (**a, c, e**) and S-wave (**b, d, f**) velocity models. The sinusoidal velocity anomalies are ±5% relative to the final models in all cases. Gaussian noise with standard deviations of 10 ms (**a, b**), 50 ms (**c, d**), and 100 ms (**e, f**) was added to the synthetic picks to reflect the different pick uncertainties for the P- and S-wave datasets. The starting models are shown on the left panels and the recovered models on the right. The recovery of velocity anomaly for Vp shows the upper crust has a resolution of 5 × 1 km (x=0-200km) and 5 × 1.5 km (200-384km), the lower crust of 5 × 2.5 km and the upper mantle of 10 × 5 km. The north-south differences in resolution in the upper crust result from the inclusion of a higher quality 20 s downward continued OBS records with a shot sampling of 50 m in the inversion at the model distance x=0-250 km and a 60 s downward continued OBS records with a shot sampling of ~150 to 190 m at the model distance x=250 to 384 km [1]. For Vs, the crust has a resolution of 5 × 2.5 km to 10 × 5 km and the upper mantle of 15 × 5 km. Increasing the gaussian noise level affects more the recovery of those small-scale anomalies (5 × 1 km, 5 × 1.5 km) and for large-scale anomalies, the differences are only subtle. To compare the size of interpreted anomalous Vp/Vs features in Fig. 2 to the size of checkers, the black dashed lines outlined these bodies are added. As finer details of Vp are "downsampled" to match Vs resolution when computing Vp/Vs ratio. Therefore, we quote the lower resolution of Vs model to be the resolution of Vp/Vs model. BT—Barracuda Trough; BR—Barracuda Ridge; TB—Tiburon Basin; DAP—Demerara Abyssal Plain.

Supplementary Fig. 16

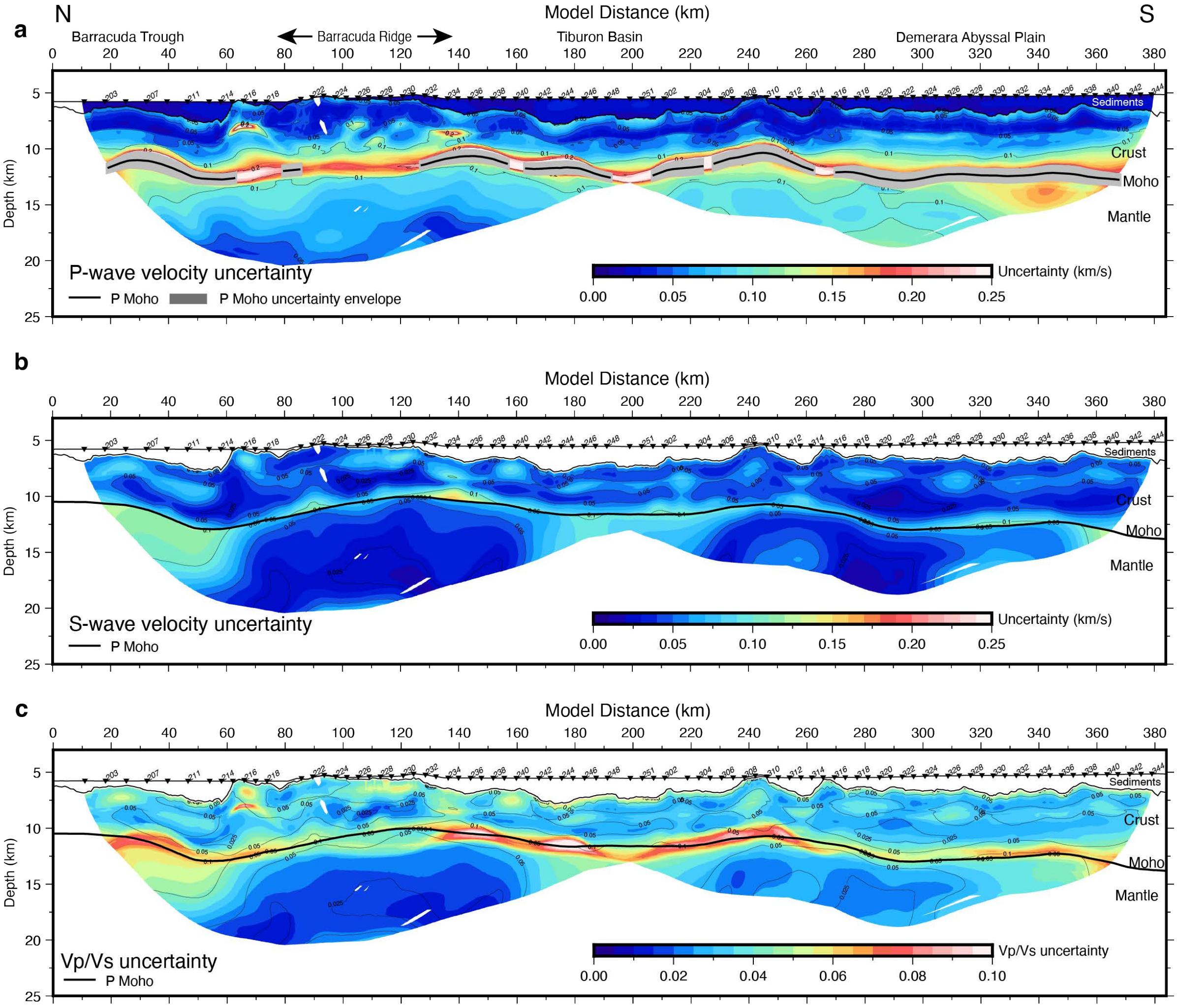

Supplementary Fig. 16. Monte Carlo velocity uncertainty in the final averaged velocity models, taken as the standard deviation of the 100 final models. a The velocity uncertainty in the P-wave model. The standard deviation of the Moho reflector is indicated with the light grey envelope. The solid black line is the average Moho reflector from 100 final models. **b** The velocity uncertainty in the S-wave model. Note that the Moho reflector used in the S-wave starting models during Monte-Carlo analysis was fixed and the final P Moho was adopted. **c** The uncertainty of Vp/Vs ratio by combining 100 different P- and S-wave models randomly and calculating its RMS misfits.

Supplementary Fig. 17

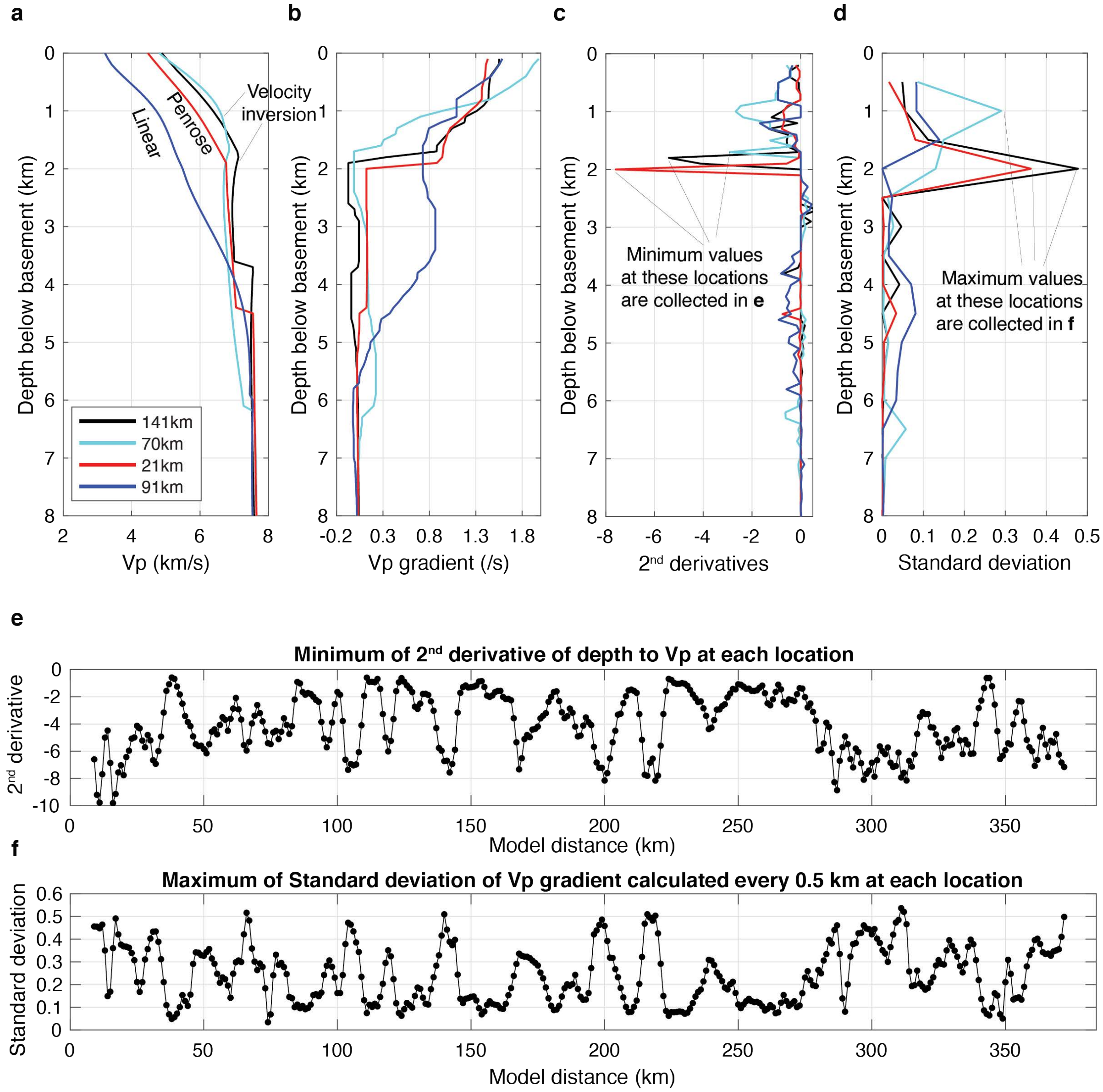

Supplementary Fig. 17. Four categories of features input to K-Means cluster analysis for P-wave velocities. a Velocity-depth profiles extracted at selected locations along the line with distinct characters. **b** The corresponding velocity gradient at these locations averaged over every 100 m depth sliding window. **c** The second derivative of depth with respect to velocity at these locations calculated every 100 m in depth range. **d** The standard deviation of velocity gradient in (**b**) statistically calculated every 500 m in depth range to avoid the effect of the variation of upper crustal thickness along the line. **e** The minimum value of the second derivatives shown in (**c**) for all locations along the line separated every 1 km apart. **f** The maximum value of the standard deviation shown in (**d**) for locations along the line separated every 1 km apart.

Supplementary Fig. 18

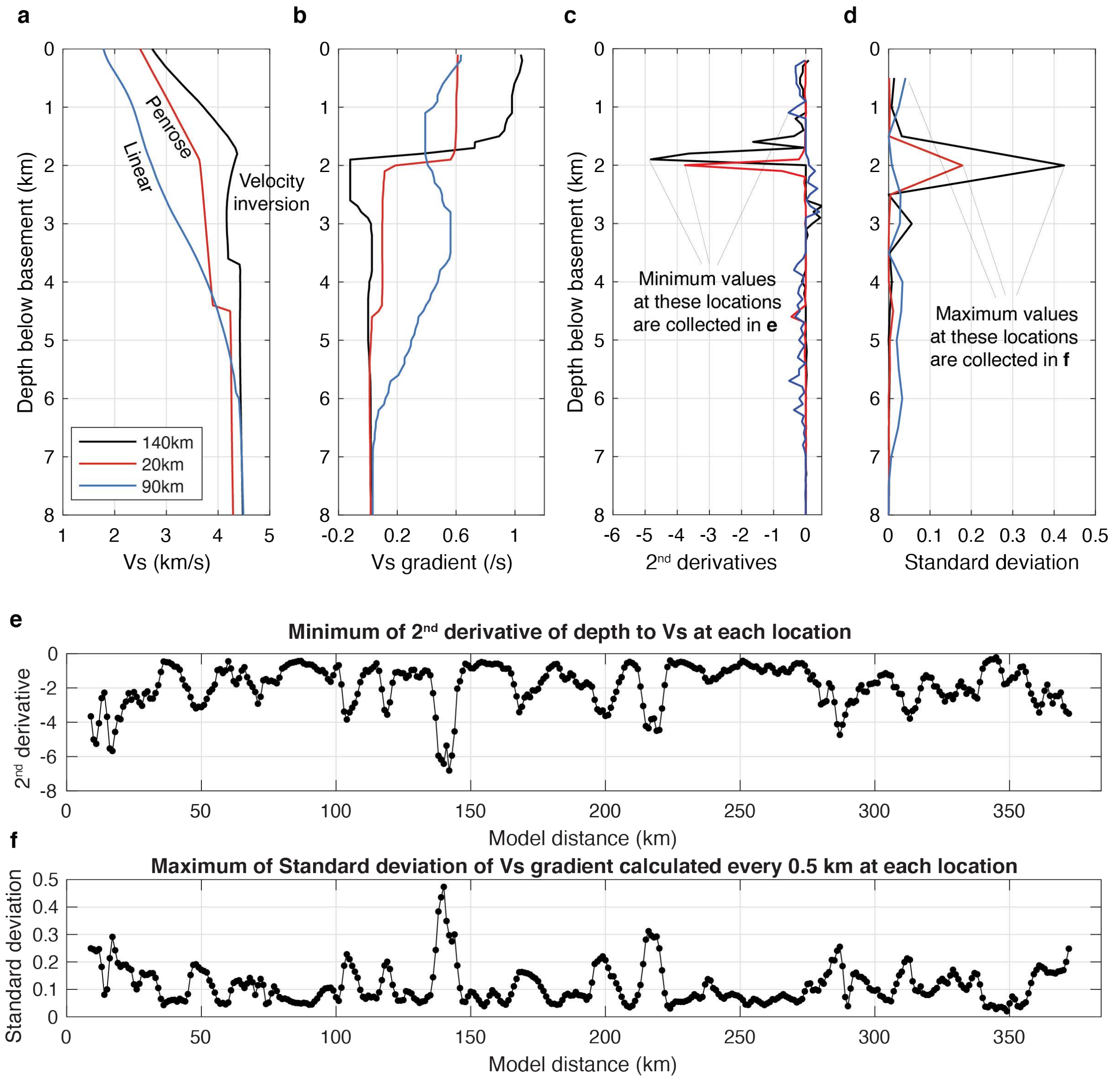

Supplementary Fig. 18. Four categories of features input to K-Means cluster analysis for S-wave velocities. The same layout of plots is shown in Supplementary Fig. 17. See the caption of Supplementary Fig. 17 for details.

Supplementary Fig. 19

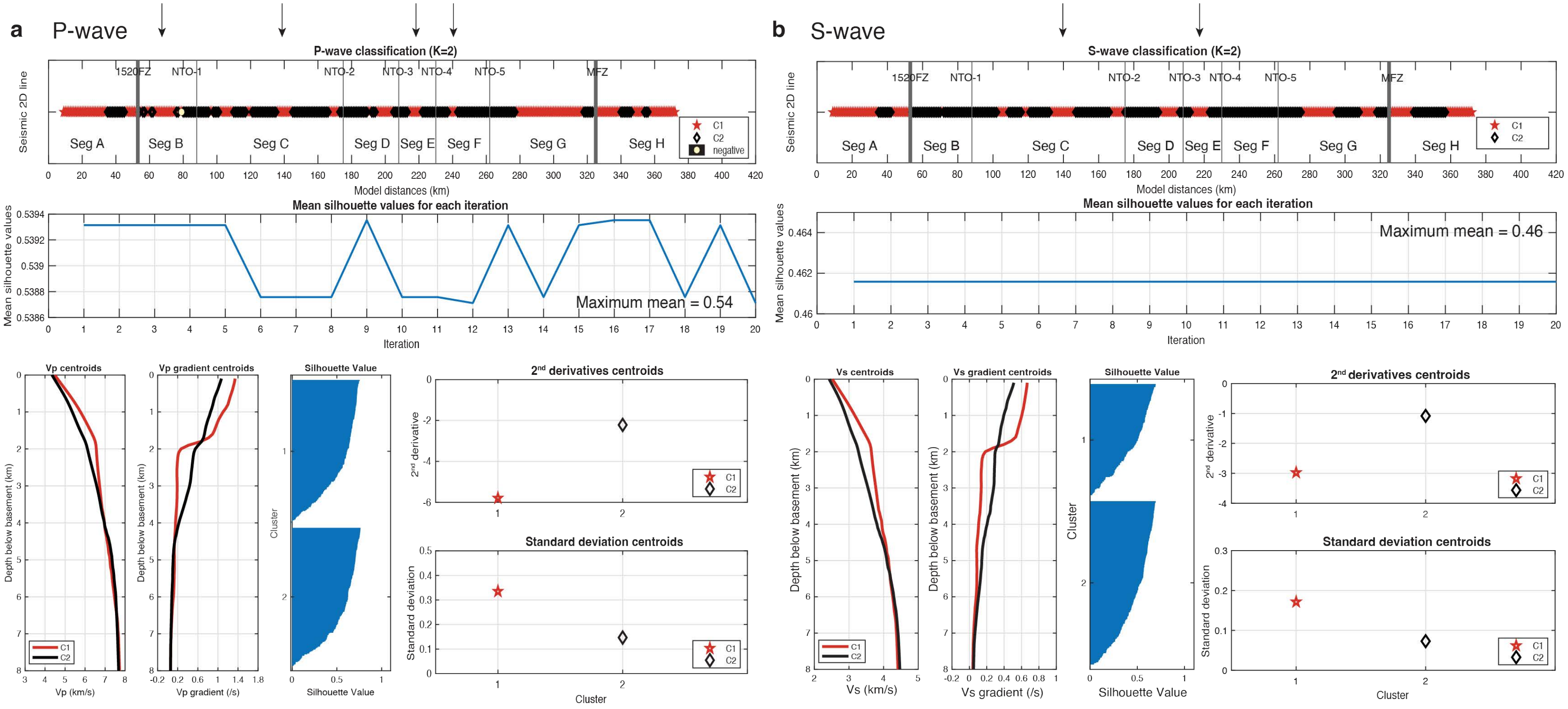

Supplementary Fig. 19. K-Means solution for two clusters (K=2) using P-wave velocities (a) and S-wave velocities (b). In each section, the upper panel is the cluster solution along the seismic line. Black arrows are locations of prominent velocity inversions observed from the Vp and Vs models (Fig. 2a, b). The middle panel is the mean silhouette value for each iteration. Silhouette value measures how similar a data point is to its own cluster compared to others. It has a range between -1 and 1. A positive value approaching to 1 means the data point is well-clustered and distinct from the other clusters. A negative value approaching -1 means the data point might be in the wrong cluster. 0 means the data point is on the boundary between clusters. The yellow asteroids shown in the upper panel are the ones that have negative silhouette values which were removed from further analysis. The variation of silhouette value for all the runs can be considered as a criterion to judge the robustness of the K-Means model. A small variation of this value suggests the model can find the same cluster solution no matter how random the initialization of the cluster centroids is. The bottom panel shows the centroids for the four categories of features and silhouette plot of the cluster solution with the maximum mean silhouette value. Note velocity inversions are mixed with that of two-layer seismic structures and thus not identifiable.

Supplementary Fig. 20

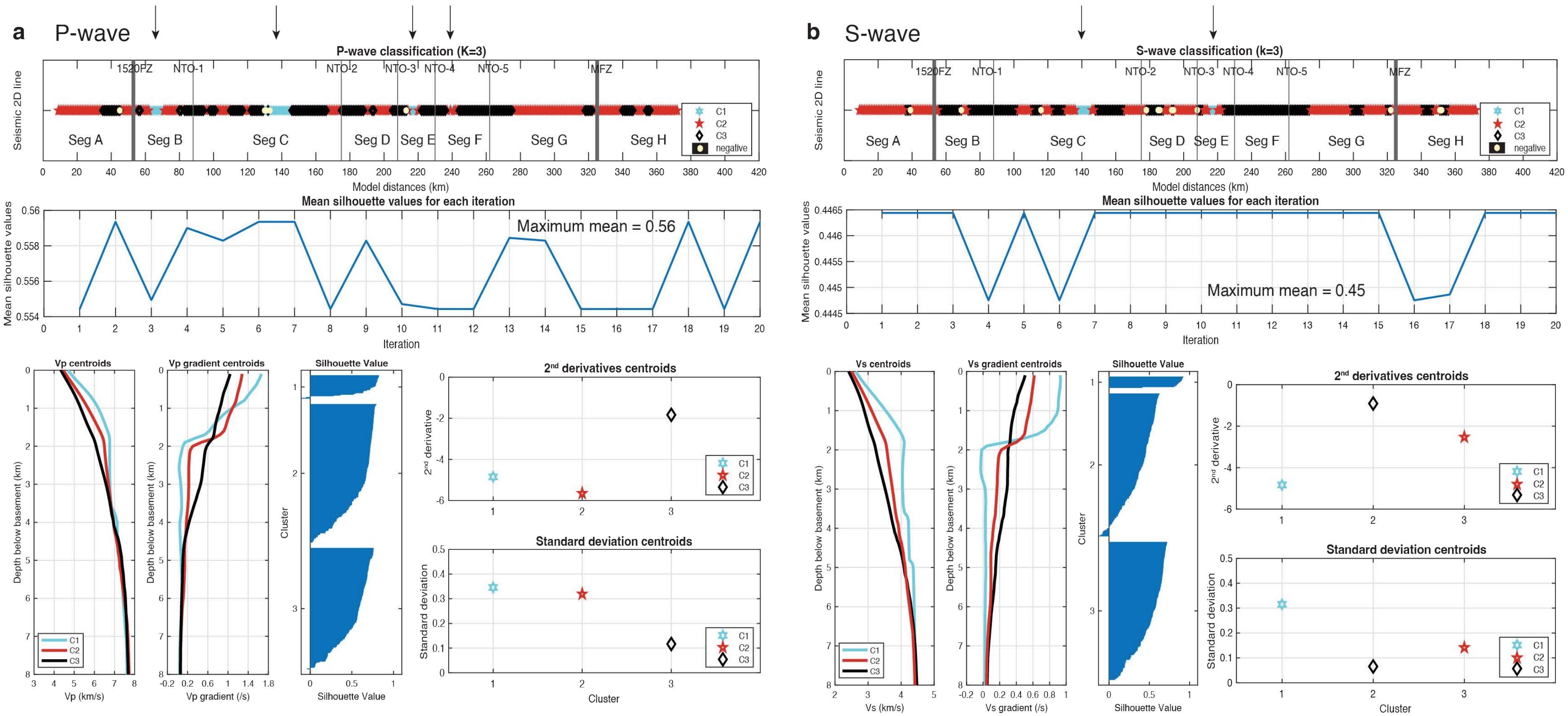

Supplementary Fig. 20. K-Means solution for three clusters (K=3) using P-wave velocities (a) and S-wave velocities (b). The same layout of plots is shown in Supplementary Fig. 19. See the caption of Supplementary Fig. 19 for details. Note that velocity inversions can be identified in this solution.

Supplementary Fig. 21

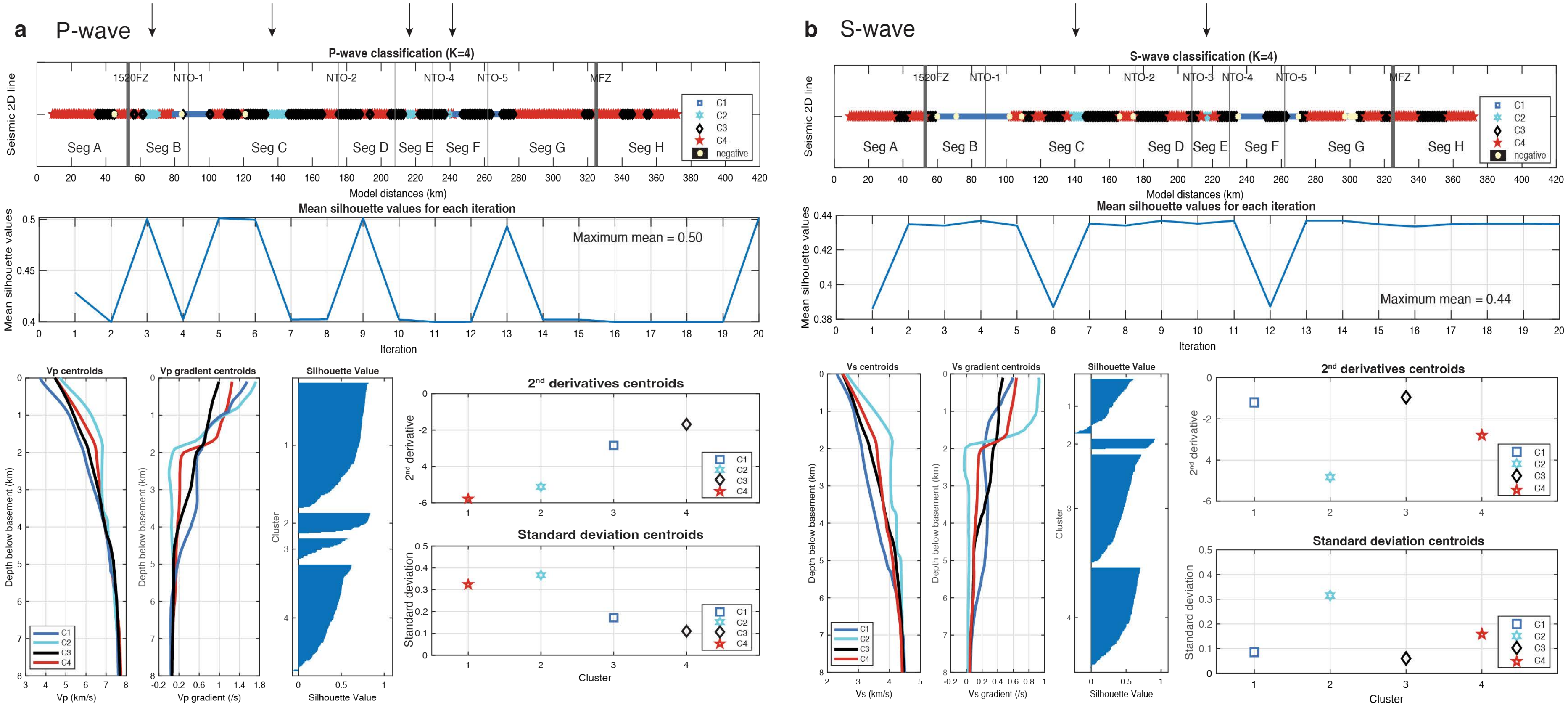

Supplementary Fig. 21. K-Means solution for four clusters (K=4) using P-wave velocities (a) and S-wave velocities (b). The same layout of plots is shown in Supplementary Fig. 19. See the caption of Supplementary Fig. 19 for details. Note that this is our preferred solution and compared to k=3 solution, a separate cluster with the lowest velocity and gradient is identified at Segments B, C and F across NTO-1 and NTO-5.

Supplementary Fig. 22

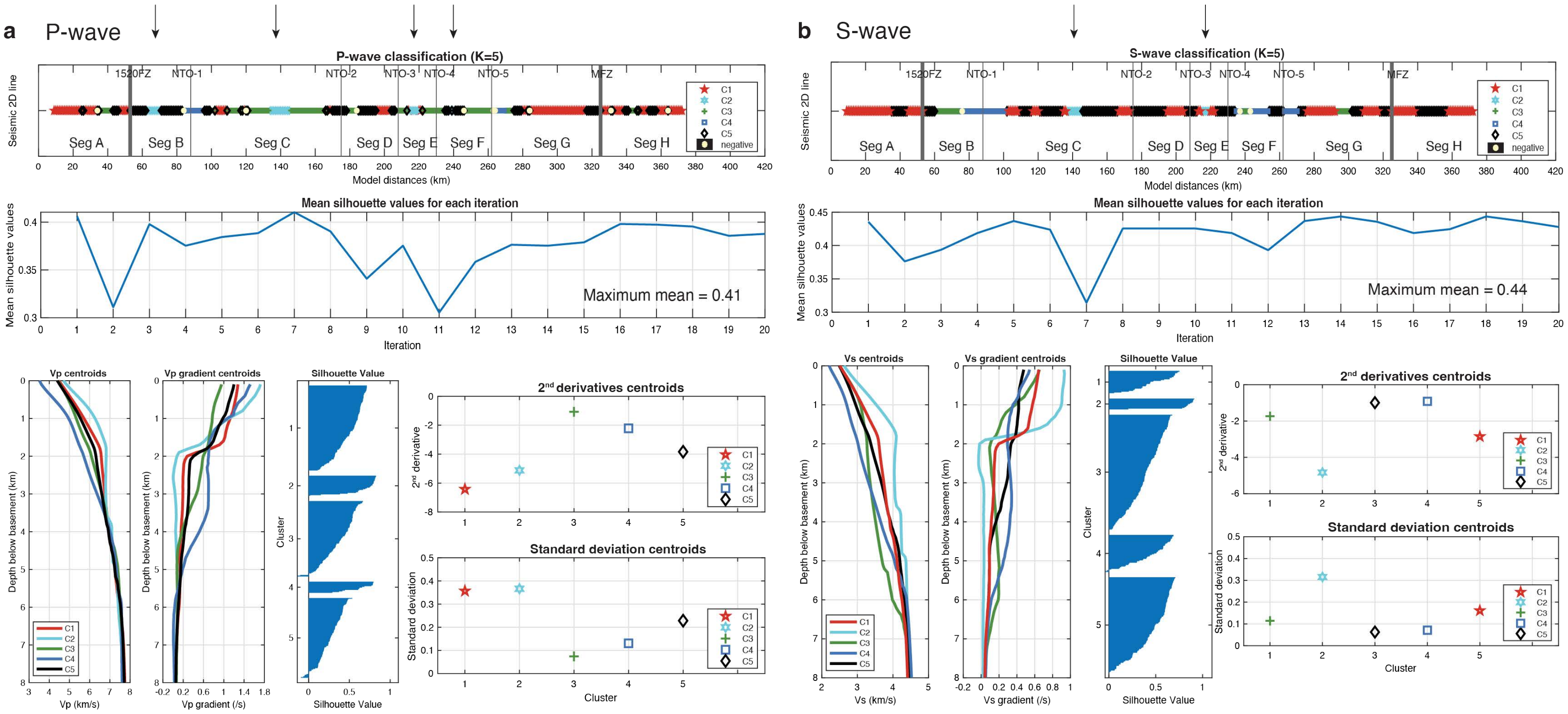

Supplementary Fig. 22. K-Means solution for five clusters (K=5) using P-wave velocities (a) and S-wave velocities (b). The same layout of plots is shown in Supplementary Fig. 19. See the caption of Supplementary Fig. 19 for details. Note how compared to the preferred k=4 solution, duplicated clusters with rather similar structures (C1 and C5 for P-wave, C3 and C4 for S-wave) are shown.

Supplementary Fig. 23

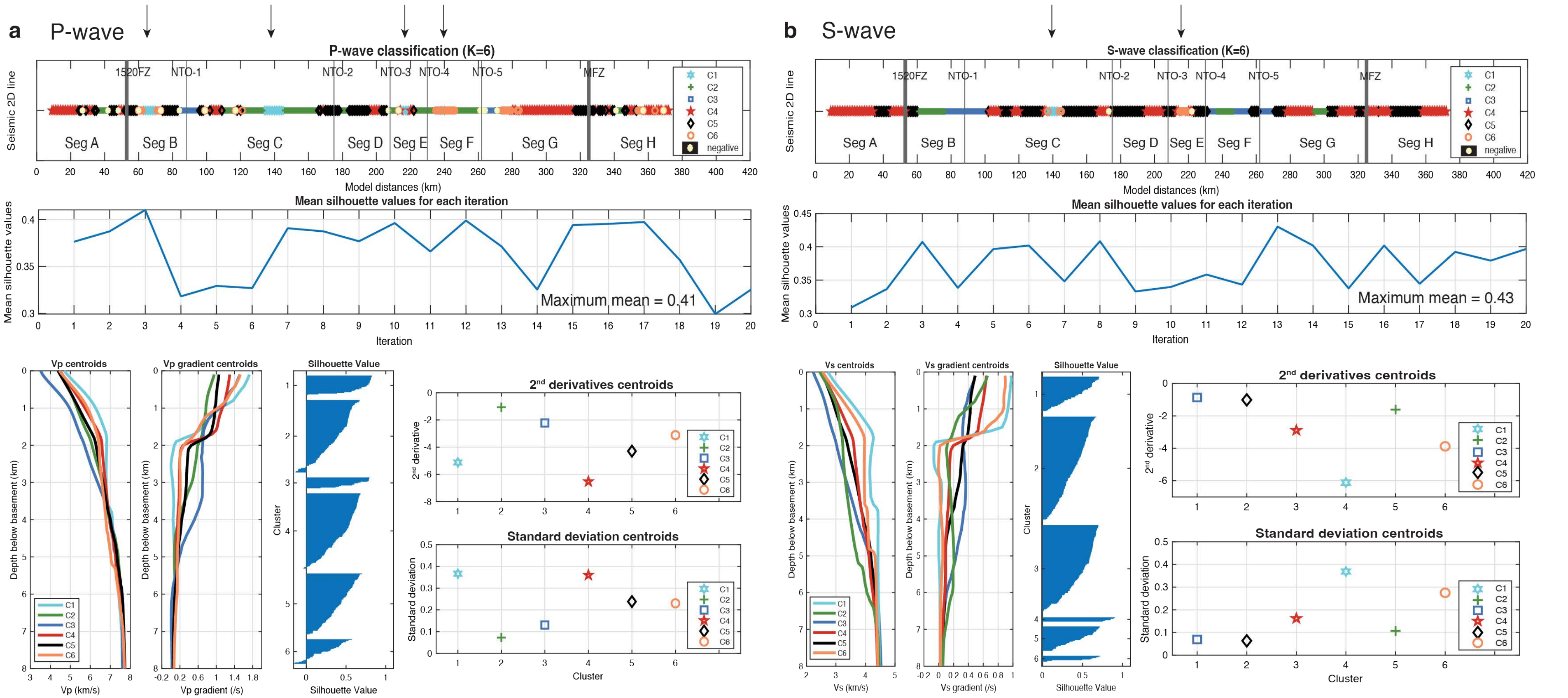

Supplementary Fig. 23. K-Means solution for six clusters (K=6) using P-wave velocities (a) and S-wave velocities (b). The same layout of plots is shown in Supplementary Fig. 19. See the caption of Supplementary Fig. 19 for details. Note how compared to the preferred k=4 solution, duplicated clusters with rather similar structures (C4 and C6, C2 and C5 for P-wave, C1 and C6, C2 and C3 for S-wave) are shown.

Supplementary Fig. 24

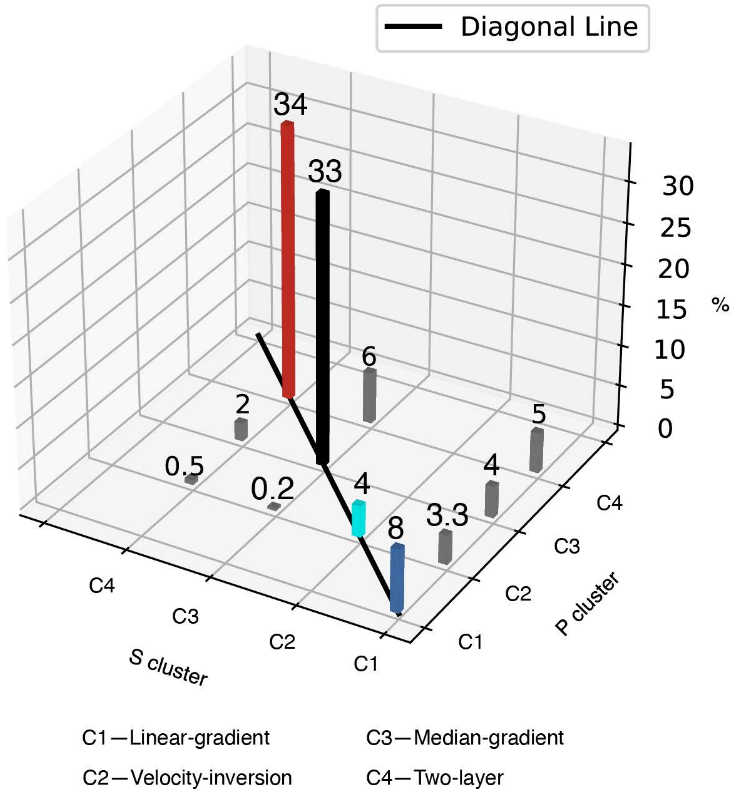

Supplementary Fig. 24. The distribution for the combinations of our preferred P- and *S*-wave clusters (K=4). This plot effectively visualises the relationship between cluster categorisation using P- and S-wave when k=4 (Supplementary Fig. 21). X-axis and Y-axis are four categories of S- and P-wave clusters. Z-axis represents the percentage of all observations corresponding to each combination of P- and S-wave clusters. We have in total 364 observations (covered by rays) of velocity attributes extracted along the seismic line. The higher the percentage (values on top of each bar), the more frequently the combination of P- and S-clusters occurs. In total, 79% of the observations (summation of values on top of bars on the black diagonal line) have velocity attributes classified into the same cluster for P- and S-waves. This suggests high consistency between the P- and S-wave velocity structures. The bars falling above or below the diagonal line (21%) are locations where different velocity attributes are grouped into different clusters for P- and S-wave. However, except at 67 and 240km (Fig. 3a), these locations are mostly located within minimal ranges, which should not affect our interpretation of magma-robust and magma-poor segments.

Supplementary Fig. 25

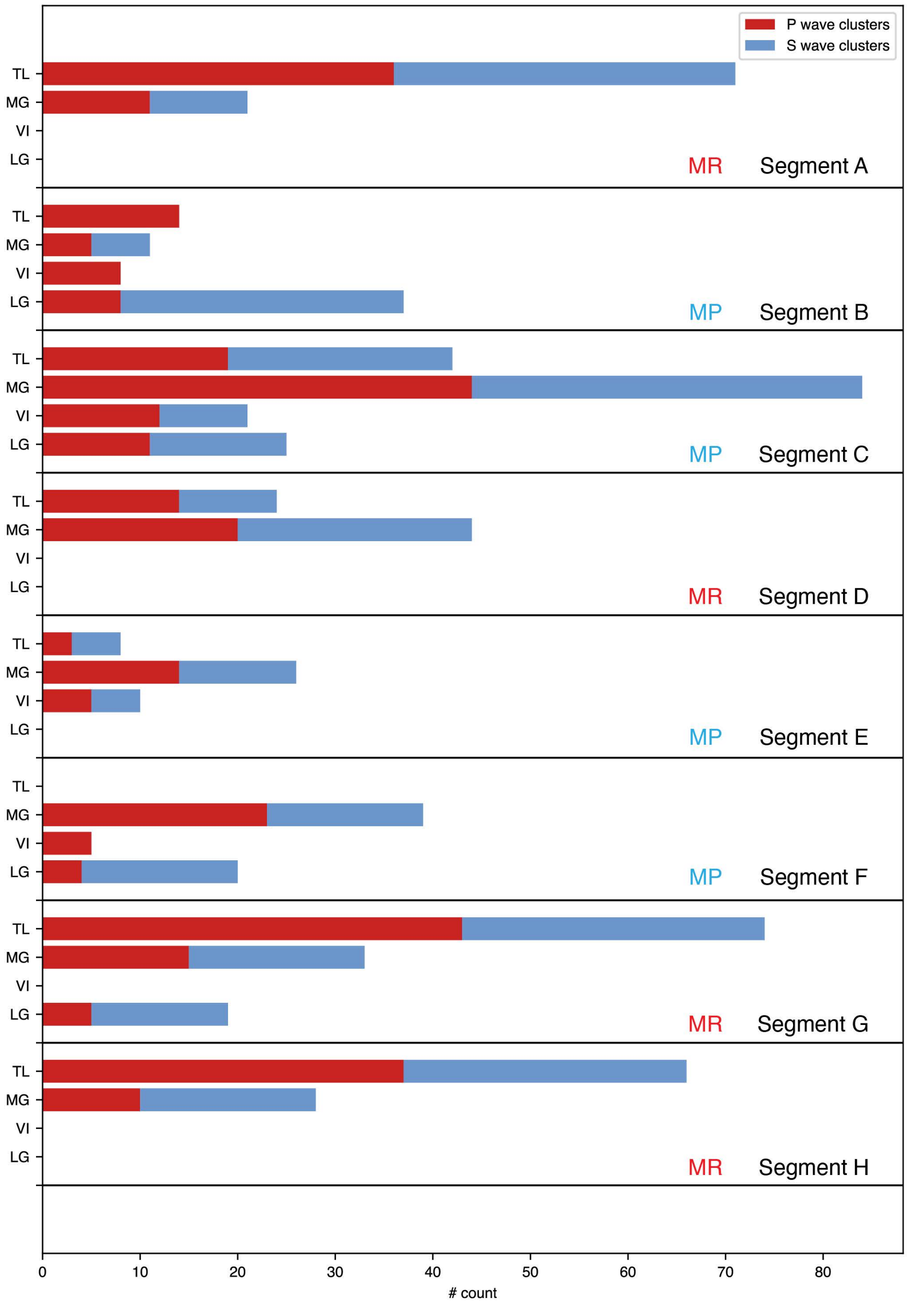

Supplementary Fig. 25. Classification of magma-robust (MR) and magma-poor (MP) segments based on the frequency of occurrence for each cluster within segments. The X-axis shows the count of occurrences for each cluster shown on the Y-axis. TL—Two-layer; MG—Median-gradient; VI—Velocity-inversion; LG—Linear-gradient. See main text for the definition. Note the segment is classified as magma-robust if the Two-layer is more dominant or only the Two-layer and Median-gradient are included. The segment is magma-poor if it mainly includes Velocity-inversion, Median- and Linear-gradient clusters, with the latter two clusters more dominant.

Supplementary Fig. 26

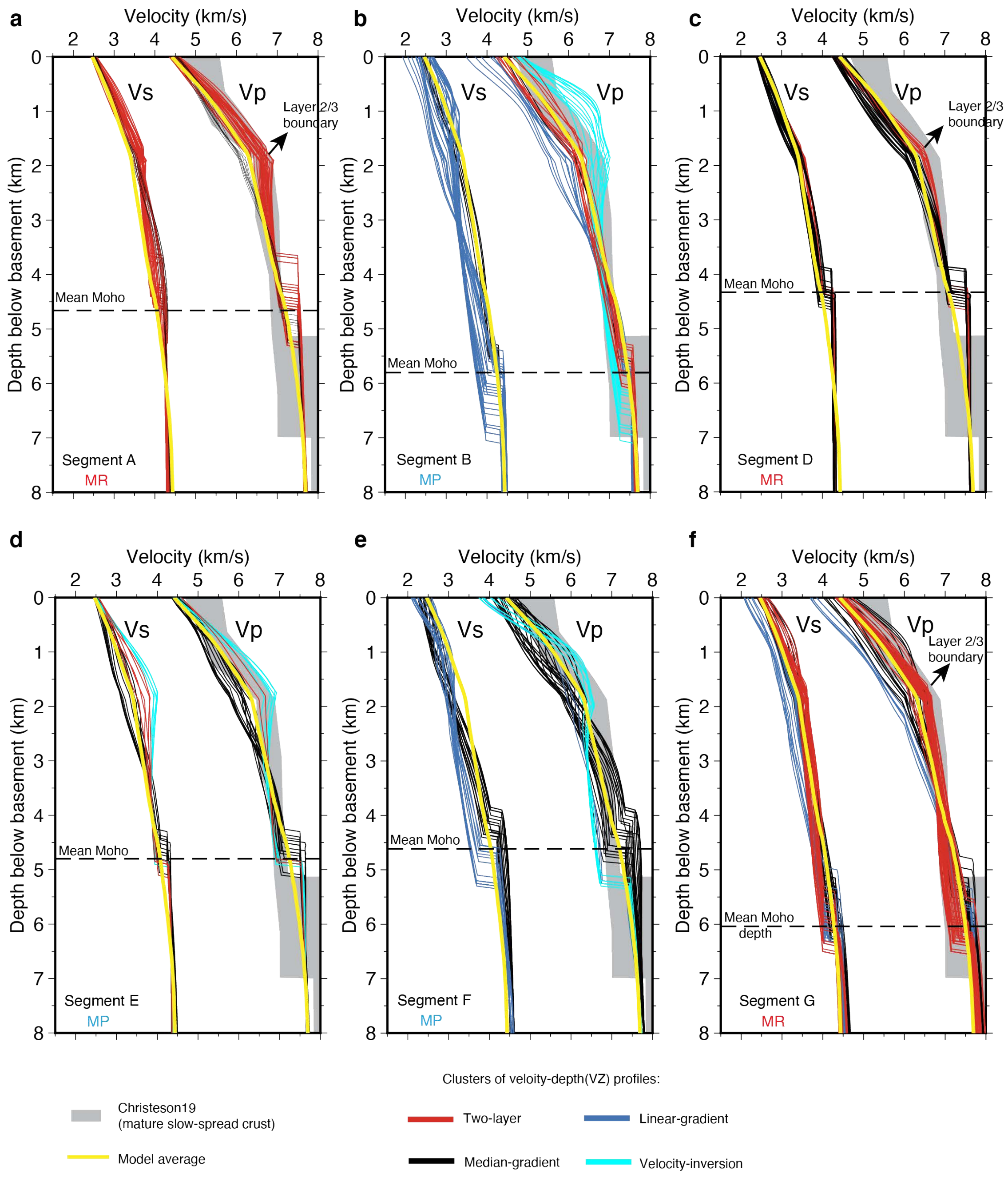

Supplementary Fig. 26. Clusters of velocity-depth (VZ) profiles for Segments A, B, D, E, F and G. Thin solid colour lines are 1-D VZ profiles for the four clusters (see main text for the definition). The thick yellow line represents the average through the entire P-wave and S-wave velocity models. Grey shade is the velocity envelope from Christeson et al.[7], for mature (>7 Ma) magmatic slow spreading crust. A dashed horizontal line represents the average depth of the resolved PmP reflections. MR—Magma-robust segment; MP—Magma-poor segment.

Supplementary Fig. 27

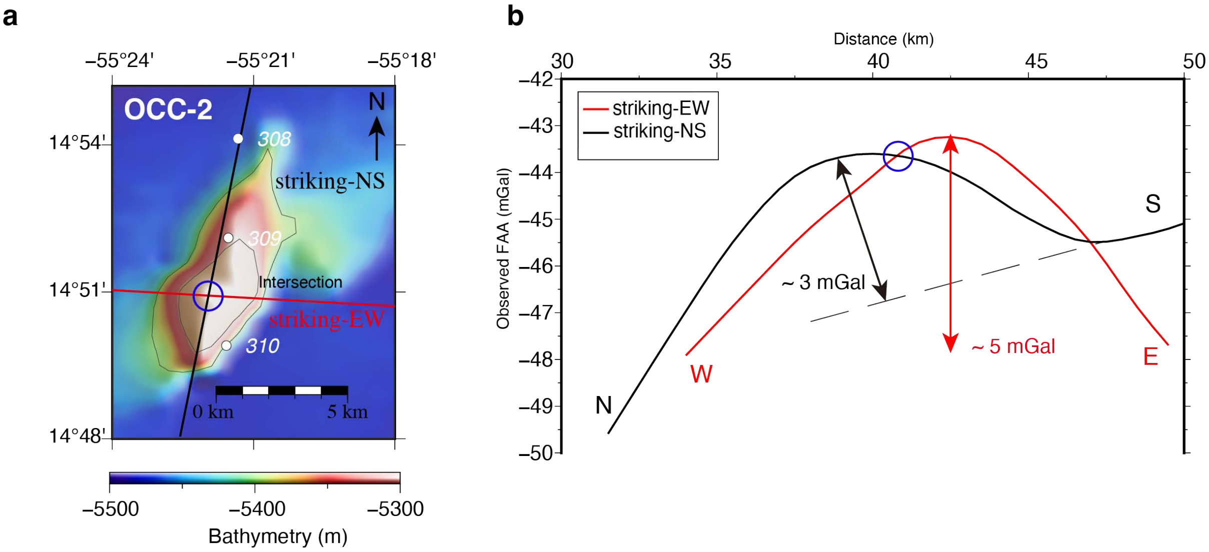

Supplementary Fig. 27. Assessment of the effect of 3D bathymetry on the gravity results. Given the water layer has the strongest effect on the computed gravity (Supplementary Fig. 28b), it is important to consider the influence of small outcropping basement highs on the results. Here the forward predicted gravity shown is computed in orthogonal directions across one of these features. Only the water layer is considered with a density contrast of 1 g/cm^3 with the sub-seabed layer. Note how the predicted 2D gravity varies by about 3 to 5 mGal in the two directions. We assigned +/- 3 mGal to indicate the uncertainty in the gravity forward modelling.

Supplementary Fig. 28

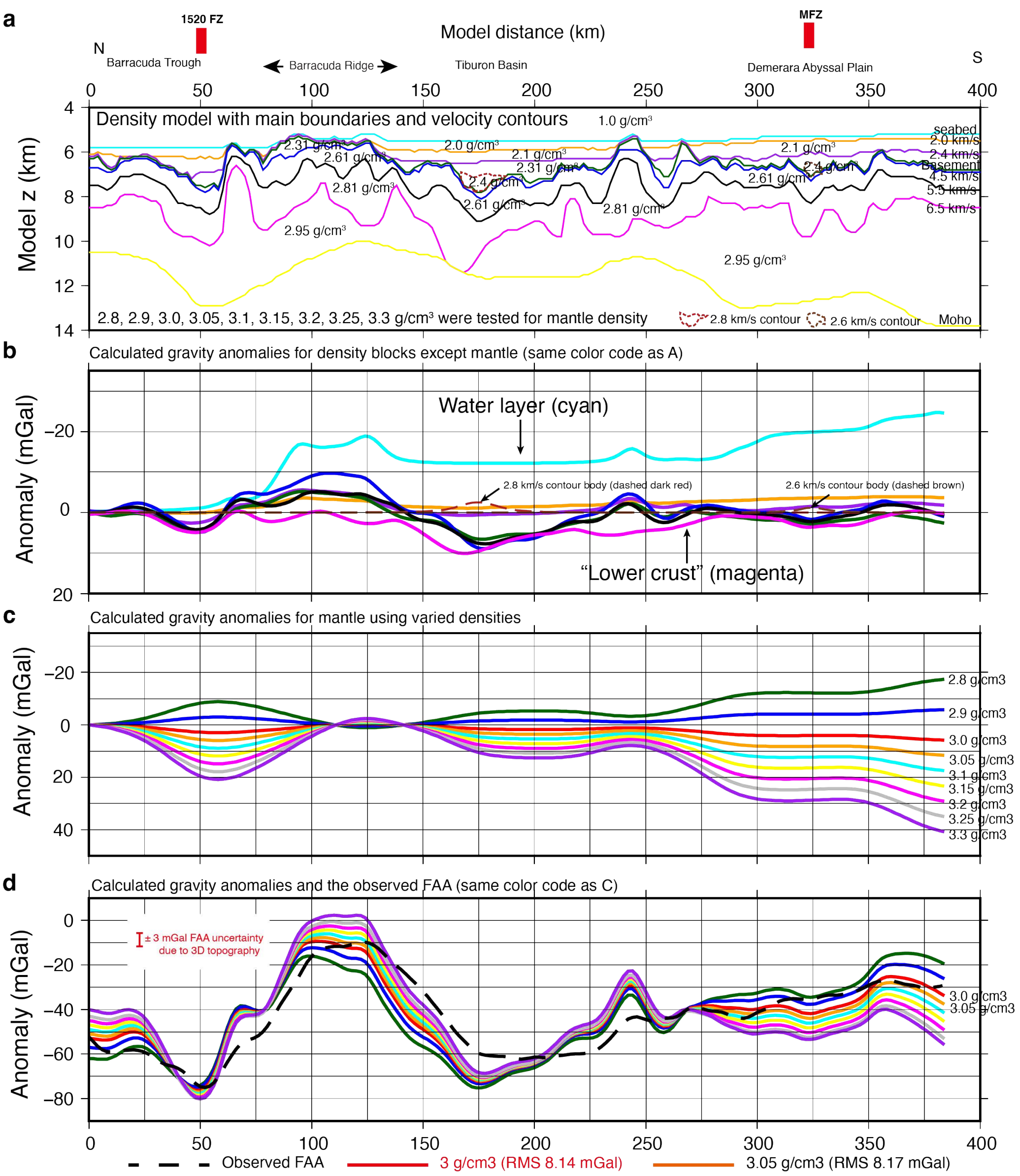

Supplementary Fig. 28. Gravity modelling using different mantle densities. **a** Density model with main physical boundaries and velocity contours extracted from P-wave velocity model. Cyan—seabed; orange—2.0 km/s contours; purple—2.4 km/s contours; dark green—basement top; blue—4.5 km/s; black—5.5 km/s; magenta—6.5 km/s; yellow—seismically determined Moho boundary. Two dashed contoured bodies (dark red and brown) in the sediments were determined by 2.8 km/s and 2.6 km/s velocity contours at the deepest of Tiburon Basin (175 km) and at Marathon Fracture Zone (MFZ: 325 km). **b** Calculated gravity anomalies for each density block with the same colour code employed as (**a**). It is noted that shallow structures have more contributions on the final gravity estimation. **c** Calculated gravity anomalies for the mantle density block using densities of 2.8 g/cm^3 to 3.3 g/cm^3. **d** Observed FAA (black dashed line) and calculated gravity anomalies (coloured solid lines) by the summation of gravity anomalies from all density blocks shown in (**b**) and (**c**). The same colour code for the gravities calculated using different mantle densities were employed as that in (**c**). Mantle density of 3.0 g/cm^3 has the smallest RMS misfits with the observed free-air gravity anomaly (FAA) along the line. 1520 FZ—Fifteen-Twenty Fracture Zone.

Supplementary Fig. 29

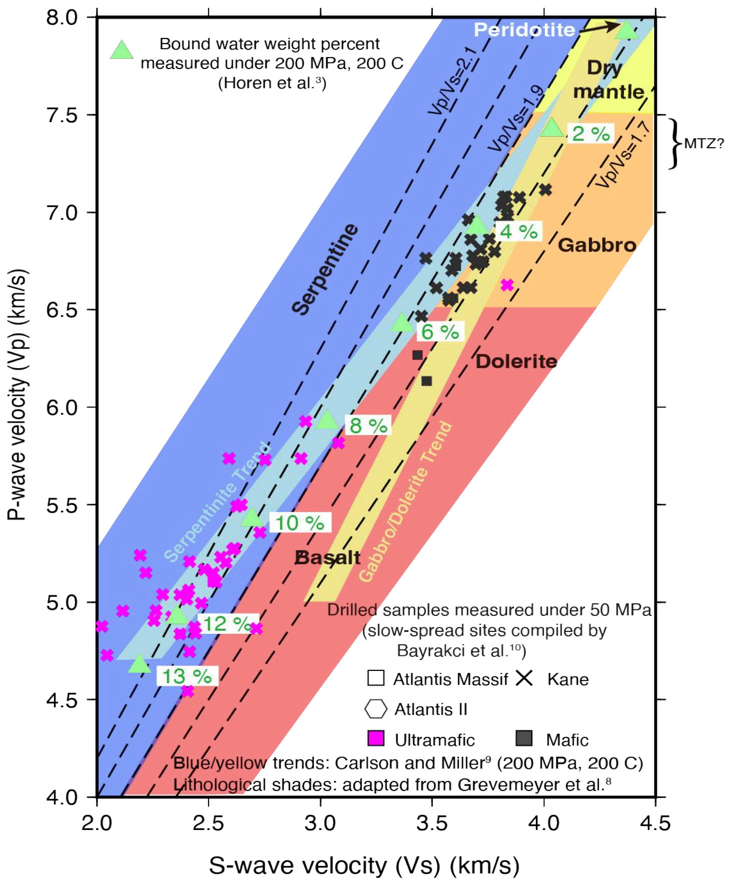

Supplementary Fig. 29. Vp and Vp/Vs ratio as a proxy for the discrimination of rock type and the identification of ultramafic rocks serpentinisation. Background coloured areas indicate bounds used to distinguish rock types in this study which is adapted from Grevemeyer et al. [8] (see "Methods" section). Laboratory measurements from samples at appropriate pressures from which the rock type bounds are based are shown. The blue trend lines is for serpentine with bound water content indicated with green triangles [3] (see values in Supplementary Table 2). The yellow trend line is for gabbro/dolerite [9]. Magenta and dark grey data points are individual measurements on samples collected by deep ocean drilling at slow-spreading sites indicated in Bayrakci et al. [10]. A Vp between 7.1 and 7.5 km/s may indicate a transition zone between gabbro and mantle peridotites [11]. As mafic and dry ultramafic rocks would not affect the estimation of the excess bound water content due to serpentinisation, for simplicity, we extended the classification of gabbroic rocks to a Vp of 7.5 km/s to align with our seismically defined Moho interface. MTZ—Moho transition zone.

Supplementary Fig. 30

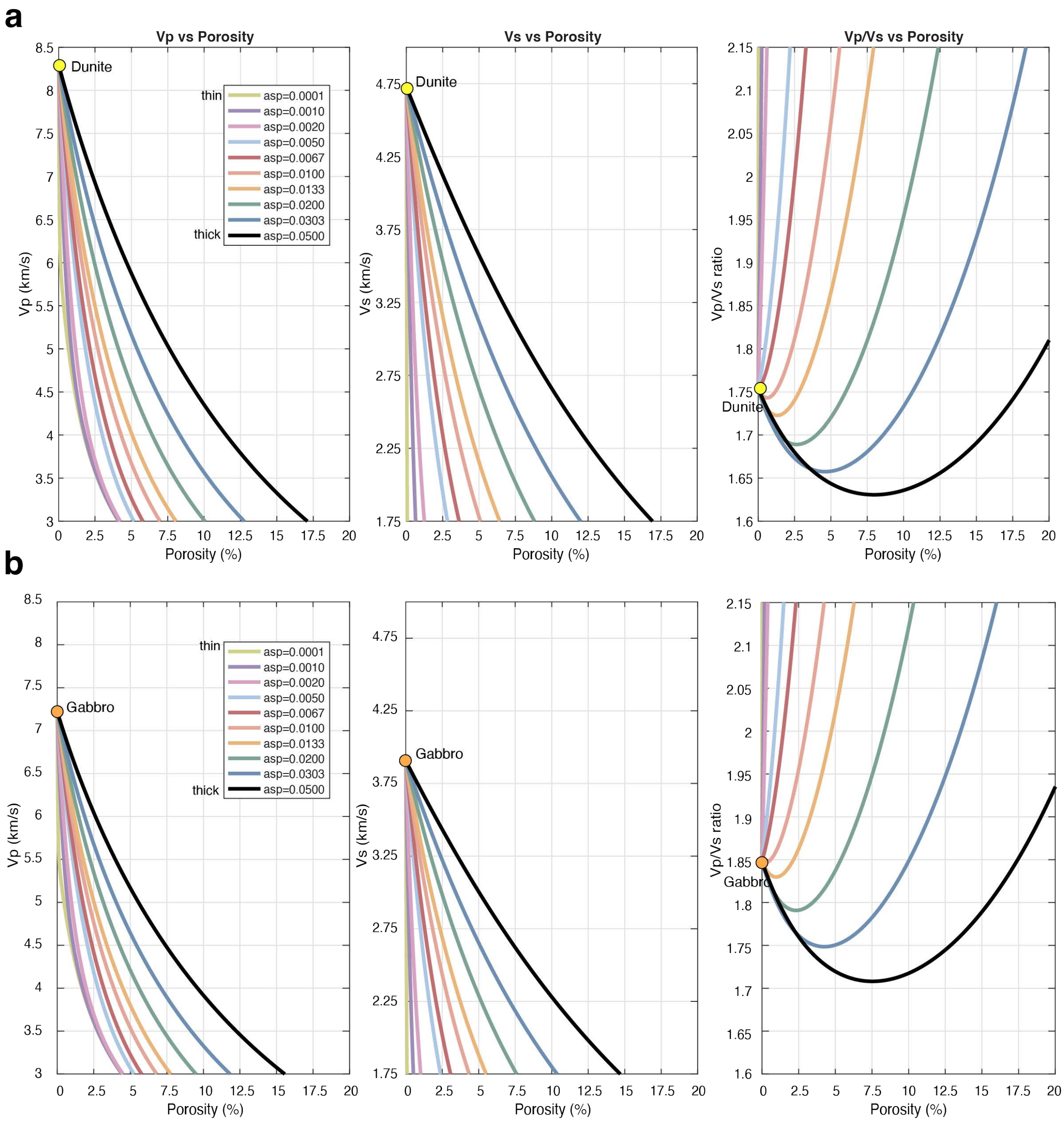

Supplementary Fig. 30. Effect of water-filled cracks on seismic velocity. Seismic P- and S-wave velocity (Vp and Vs) and Vp/Vs ratio with increasing porosity calculated using differential effective medium (DEM) theory [12,13] with inclusion of randomly oriented fractures with aspect ratios (asp) from 0.0001 to 0.05**.** We took dry dunite [4] (**a**) with Vp of 8.299 km/s, Vs of 4.731 km/s and density of 3.310 g/cm^3 and dry gabbro [4] (**b**) with Vp of 7.138 km/s, Vs=3.862 km/s and density of 2.968 g/cm^3 for the host material in DEM calculations. We added increasing proportions of water-filled fractures in the scheme with water density of 1.03 g/cm^3, Vp of 1.5 km/s and Vs of 0 km/s. According to the observed ranges of Vp and Vs, the maximum porosity which is appropriate is 17.5%. The relations between Vp and Vp/Vs for a given porosity are present in Fig. 6b and Supplementary Fig. 31 for different aspect ratios, which form the basis for fracture water estimation.

Supplementary Fig. 31

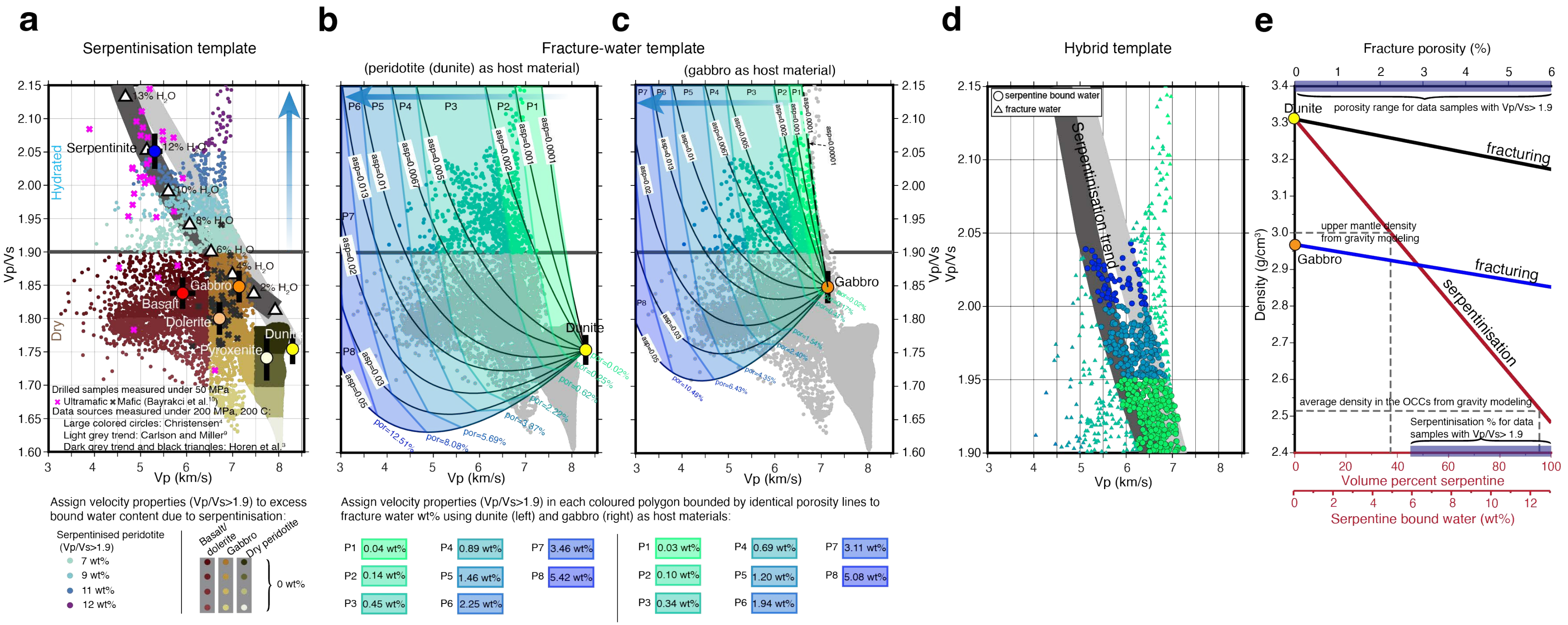

Supplementary Fig.31. Interpretation of Vp/Vs anomalies based on lithology, serpentinisation, water-filled fracturing and density. a Relationship between lithology, serpentinisation and Vp/Vs. Velocity points (small filled circles) from our preferred velocity models (Fig. 2) are projected onto the lithological template of Vp/Vs versus Vp. The definition of lithological bounds is detailed in "Methods" and is based on laboratory measurements from Christensen [4] (large coloured circles) and drilled ultramafic/mafic samples measured at 50 MPa from slow-spreading sites [10] (magenta/dark grey crosses) together with serpentinisation trends from peridotite to serpentinite (light grey—Carlson and Miller[9] ; dark grey—Horen et al.[3]) measured at 200 MPa and 200°C. White triangles indicate the bound water content from Horen et al. [3] with the blue arrow showing an increase in bound water content with the increase in Vp/Vs. Velocity points in dark red, mustard yellow and olive green are assumed standard crustal and mantle rocks and so assigned 0 wt% excess bound water content. Velocity points in blue and purple are assigned increased bound water content based on the dark grey serpentinisation trend from Horen et al.[3] (see "Methods" and

Supplementary Table 2). The classified velocity points are shown in their model positions in Fig.7a and the corresponding excess bound water content model is shown in Fig. 7b. **b** and **c** Relationship between water-filled fractures and Vp/Vs. Note that the template is calculated using DEM when including fractures in a dry dunite (**b**) and a gabbro (**c**) host matrix. The small filled grey circles are the velocity points as shown in **a**. The black lines are Vp/Vs against Vp trend for water-filled fractures with different aspect ratios (asp) calculated from differential effective medium (DEM) analysis [12,13] (see "Methods") with dry dunite and dry gabbro as the host material. The coloured polygons delineate regions with different fracture porosities (and hence water content) as indicated (Supplementary Table 3). The blue arrow indicates the increase in fracture water with the decrease in Vp. The classified velocity points (Vp/Vs > 1.9) are shown in their model positions together with the converted fracture water content in Supplementary Fig. 32b and c. Note the range of aspect ratios can cover all velocity points (grey small circles in the background in **b** but even with much thinner cracks (aspect ratio=0.00001), the fracture-water template based on a gabbro matrix cannot fully cover the velocity points with Vp/Vs>1.9 and Vp of ~ 7 km/s in **c**. Note that water-filled fractures are unlikely in mature crust buried by sediment[7], but these models are included for completeness. **d** A hybrid template of serpentine bound (filled circles) and fracture-water (filled triangles) for estimating water content for velocity points with Vp/Vs > 1.9. Serpentinisation trend same in **a**. Water content is assigned by following **a** and **b** for bound or fracture water, which converts Vp/Vs to the hybrid hydration model in Supplementary Fig. 32d. **e** Change in bulk density associated with increased porosity of dunite/gabbro (top axis) and increased serpentinisation degree of dunite (bottom axis). Note that the density reduction associated with the range of fracture porosities applicable to velocity points with Vp/Vs>1.9 is insufficient to account for the density drop modelled from gravity (Fig. 4). However, this density reduction is easily achieved by serpentinisation at levels indicated by velocity points with Vp/Vs > 1.9. Note that **a**, **b** and **e** are present in Fig. 6.

Supplementary Fig. 32

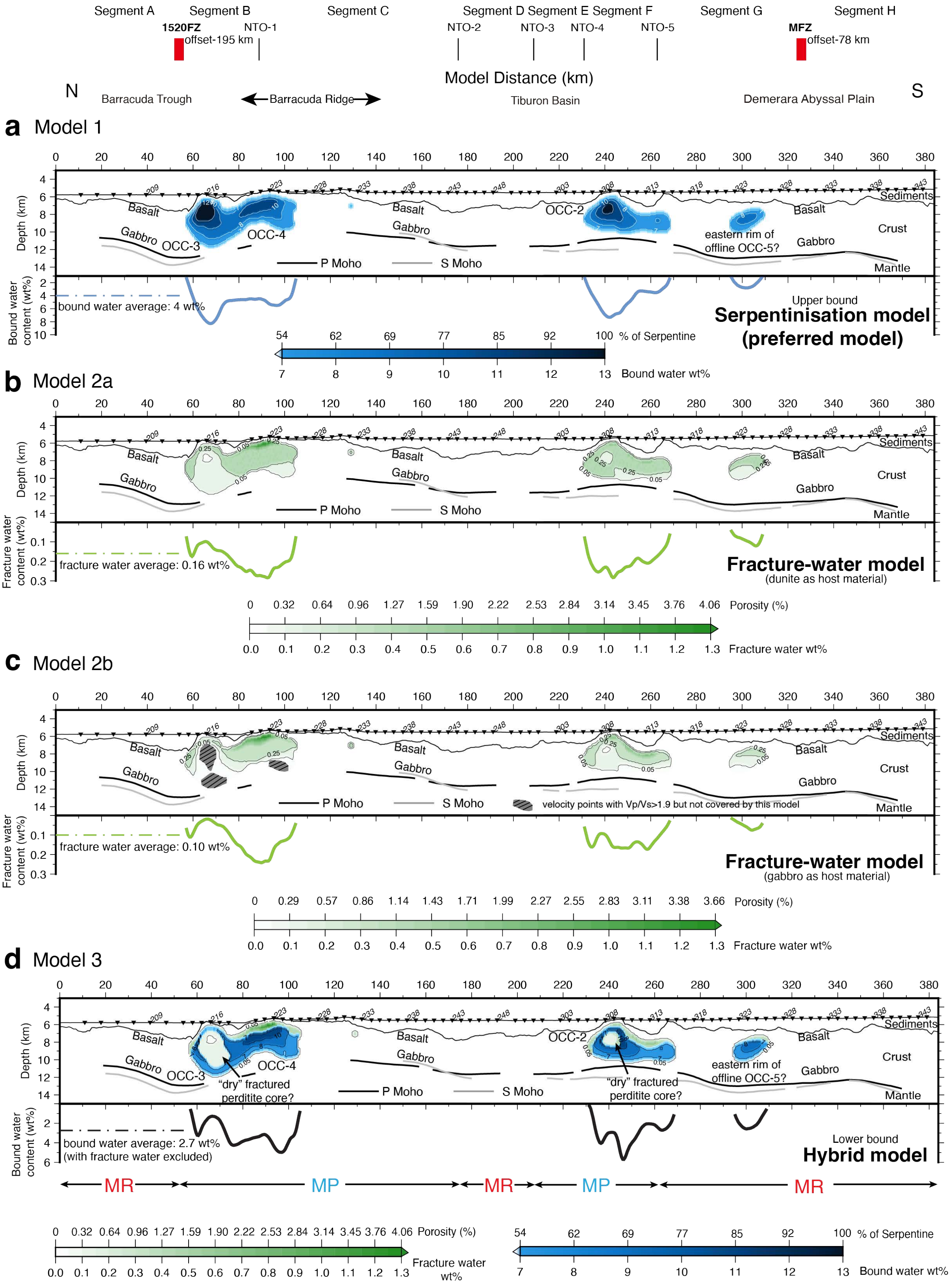

Supplementary Fig. 32. Different models considered to estimate the hydration of interpreted OCCs (regions with Vp/Vs > 1.9). a Our preferred excess hydration model where Vp/Vs> 1.9 is interpreted as serpentinite. The excess bound water due to

serpentinisation is assigned based on Supplementary Fig. 31a. **b** The fracture-water model with the dunite matrix (Supplementary Fig. 31b). **c** Same type of model as in **b** but using a gabbro matrix (Supplementary Fig. 31c). Note that the grey shaded areas are not covered by the template even with extremely thin cracks. **d** The hybrid model shows water content stored in fractured peridotite (green shades) and bounded in serpentinites (blue shades). The water content is assigned based on the hybrid template in Supplementary Fig. 31d. Note that this model takes fractured peridotite as an example to calculate the fracture water. Overall, the average hydration estimated in the OCCs using different models is 4, 0.16, 0.10 and 2.7 wt%, respectively. See "Methods" for details on generating these models.

Supplementary Fig. 33

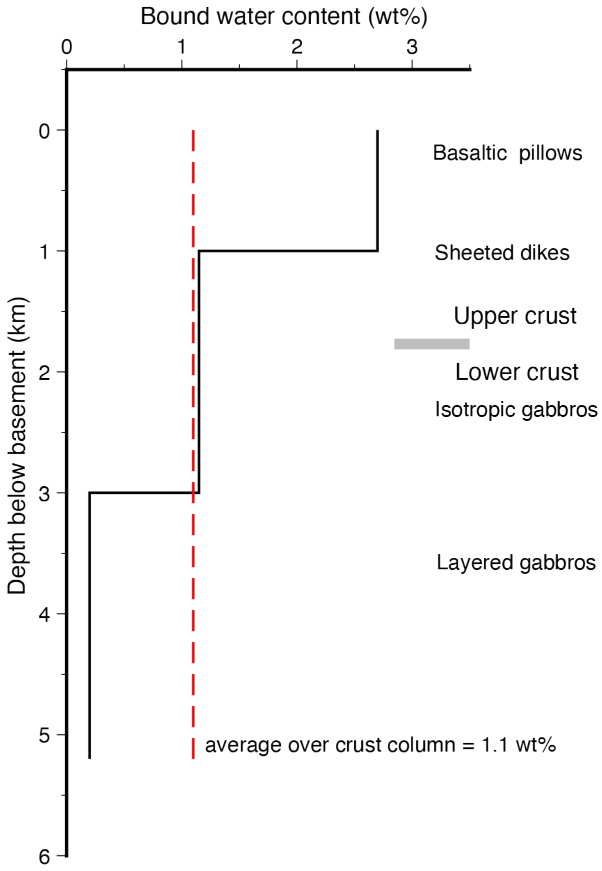

Supplementary Fig. 33. Hydration of oceanic crust with a Penrose stratigraphy adapted from Rüpke et al.[14]. The red dashed line is the average water content (1.1 wt%) through the whole crustal column, which is used in calculations of water flux for a Penrose crustal model discussed in the main text. To compare with our results, the crustal thickness is modified to 5.2 km, which is the average crustal thickness for the portion of the Atlantic crust present in this study. The grey line separates the upper and lower crust and has a value of 1.77 km, which is the average thickness of seismic Layer 2 (upper crust) for magma-robust segments from this study.

References

1 Li, L., Collier, J., Henstock, T. & Goes, S. Downward continued ocean bottom seismometer data show continued hydrothermal evolution of mature oceanic upper crust. *Geology* **52**, 717-722 (2024).

2 Carlson, R. L. & Miller, D. J. Mantle wedge water contents estimated from seismic velocities in partially serpentinized peridotites. *Geophys Res Lett* **30**, 1250 (2003).

3 Horen, H., Zamora, M. & Dubuisson, G. Seismic waves velocities and anisotropy in serpentinized peridotites from xigaze ophiolite: Abundance of serpentine in slow spreading ridge. *Geophys Res Lett* **23**, 9-12 (1996).
4 Christensen, N. I. Poisson's ratio and crustal seismology. *Journal of Geophysical Research: Solid Earth* **101**, 3139-3156 (1996).
5 Korenaga, J. *et al.* Crustal structure of the southeast Greenland margin from joint refraction and reflection seismic tomography. *Journal of Geophysical Research-Solid Earth* **105**, 21591-21614 (2000).
6 Pichot, T. et al. The Cenozoic tectonostratigraphic evolution of the Barracuda Ridge and Tiburon Rise, at the western end of the North America-South America plate boundary zone. *Marine Geology* **303**, 154-171 (2012).
7 Christeson, G. L., Goff, J. A. & Reece, R. S. Synthesis of Oceanic Crustal Structure From Two-Dimensional Seismic Profiles. *Reviews of Geophysics* **57**, 504-529 (2019).
8 Grevemeyer, I. *et al.* Episodic magmatism and serpentinized mantle exhumation at an ultraslow-spreading centre. *Nature Geoscience* **11**, 444-448 (2018).
9 Carlson, R. L. & Miller, D. J. A new assessment of the abundance of serpentinite in the oceanic crust. *Geophys Res Lett* **24**, 457-460 (1997).
10 Bayrakci, G. *et al.* Anisotropic Physical Properties of Mafic and Ultramafic Rocks From an Oceanic Core Complex. *Geochemistry, Geophysics, Geosystems* **19**, 4366-4384 (2018).
11 Grevemeyer, I., Ranero, C. R. & Ivandic, M. Structure of oceanic crust and serpentinization at subduction trenches. *Geosphere* **14**, 395-418 (2018).
12 Mukerji, T., Berryman, J., Mavko, G. & Berge, P. Differential effective medium modeling of rock elastic moduli with critical porosity constraints. *Geophys Res Lett* **22**, 555-558 (1995).
13 Taylor, M. A. J. & Singh, S. C. Composition and microstructure of magma bodies from effective medium theory. *Geophysical Journal International* **149**, 15-21 (2002).
14 Rüpke, L. H., Morgan, J. P., Hort, M. & Connolly, J. A. D. Serpentine and the subduction zone water cycle. *Earth and Planetary Science Letters* **223**, 17-34 (2004).
